# Supplementary material for: viGEN: An Open Source Pipeline for the Detection and Quantification of Viral RNA in Human Tumors
Source: Front Microbiol. 2018 Jun 5;9:1172. doi: 10.3389/fmicb.2018.01172 (PMC5996193; doi:10.3389/fmicb.2018.01172)
Supplement: Additional File 1 — viGEN Github tutorial. [file Data_Sheet_1.PDF]

ICBI / **viGEN**

viGEN - A bioinformatics pipeline for the exploration of viral RNA in human NGS data

Edit

Add topics

47 commits | **1** branch | **0** releases | **1** contributor

Branch: **master** | New pull request | Create new file | Upload files | Find file | Clone or download

kb472 Update README.md — Latest commit `f9ea4dc` 19 hours ago

.DS_Store	removing files	2 years ago
README.md	Update README.md	19 hours ago
collate.output.files.Rmd	Add files via upload	2 years ago
collate.output.files.pdf	Add files via upload	2 years ago
count.reads.in.regions.Rmd	Add files via upload	2 years ago
count.reads.in.regions.pdf	Add files via upload	2 years ago
workflow.png	image	2 years ago

README.md

viGEN tutorial

viGEN is a bioinformatics pipeline for the exploration of viral RNA in human NGS data. In this tutorial, we provide and end to end workflow on how this pipeline can be used on an example data file.

Steps in general

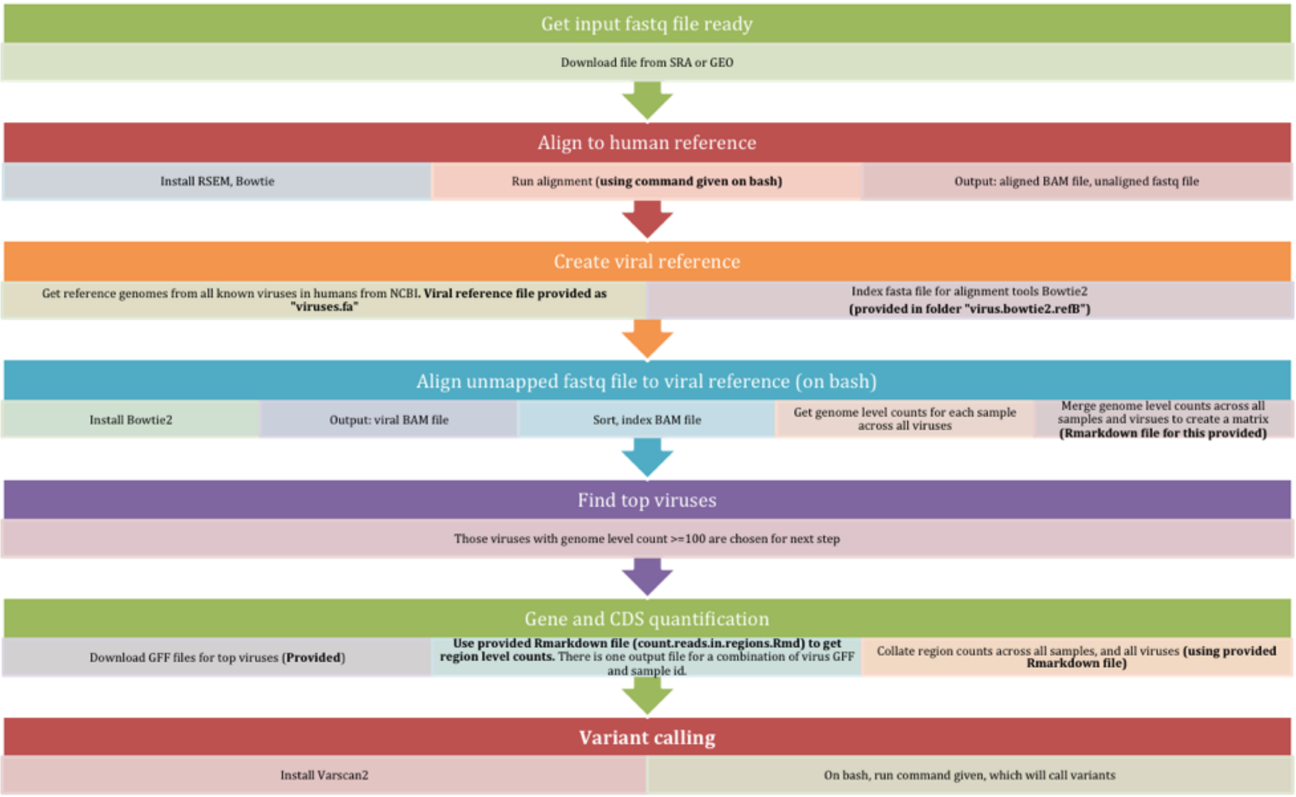

About the data

- For this tutorial, we used a sample RNA-seq file from liver cancer from public study SRA http://www.ncbi.nlm.nih.gov/bioproject/PRJNA279878 or GEO http://www.ncbi.nlm.nih.gov/geo/query/acc.cgi?acc=GSE65485 (also available at EBI SRA here: http://www.ebi.ac.uk/ena/data/view/SRR1946637).
- About the dataset: Data consists of Liver cancer patients in China. The authors look for HBV-human integration sites in the dataset of 50 Liver cancer patients in China and 5 adjacent normal tissues. We downloaded the raw reads for one sample SRR1946637 and unzipped them. This sample is from an adjacent normal liver tissue.

Workflow steps in detail

A complete summary of the input, and output files from each step in this workflow is available via google drive ***here*** *.*

Alignment to human reference

- In this tutorial, we use tool RSEM for alignment
- Install RSEM as shown in https://github.com/bli25ucb/RSEM_tutorial
- RSEM uses aligner Bowtie1, Bowtie2 or STAR. We chose to use Bowtie1 which is available as pre-compiled executable. For Bowtie1 installation, follow instructions in this manual: http://bowtie-bio.sourceforge.net/manual.shtml#obtaining-bowtie
- Test RSEM successful installation `./rsem-calculate-expression --version` and `./rsem-calculate-expression --help`
- Generate transcriptome reference for RSEM:

  RSEM uses a transcriptome reference for alignment. RSEM has a command called `rsem-prepare-reference` where it uses the genome fasta reference file and the GTF file to create the transcriptome reference. We have generated and provided these files for human hg19 reference genome in the **RSEM_reference/Rsem_ref_hg19** folder via google drive.

  If you are interested in using another reference, please follow steps provided here to generate transcriptom reference for your file: https://github.com/bli25ucb/RSEM_tutorial

- Once RSEM is installed, and the transcriptome reference is ready, run alignment using RSEM on SRA fastq files using following command: `./rsem-calculate-expression --paired-end -p 8 --output-genome-bam --keep-intermediate-files --bowtie-path /Users/username/Documents/software/bowtie/bowtie-1.1.2 --append-names --estimate-rspd --time input/SRR1946637_1.fastq input/SRR1946637_2.fastq Rsem_ref_hg19/Rsem_ref_hg19 output/SRR1946637`

  Output files: In addition to the aligned BAM file (genome level and transcriptome level), this will generate the unaligned (unmapped) fastq files named **SRR1946637_un_1.fq** and **SRR1946637_un_2.fq**. They consist of the reads that did not align to the human reference.

  This workflow is part of "Module 1 (filtered human input files)" in Figure 1 in the viGEN manuscript (currently under preparation).

Create viral reference

- We were interested in exploring all viruses existing in humans. So we first obtained reference genomes of all known and sequenced human viruses obtained from NCBI (as of Sep 2015), and merged them into one file (referred to as the "viral reference file") in fasta file format. Merge all virus fasta file into one big fasta file called `viruses.fa` . For this tutorial, we have provided this file through google drive **here**.
- We have also indexed the viral reference file, so that these files are ready for alignment tools Bowtie2 (folder name: `virus.bowtie2.refB`) or BWA (folder name: `viral.bwa.ref`) through google drive **here**.

  In case user is interested in creating a reference index the reference file on their own, this is the command to use: `./bowtie2-build /Path/viruses.fa virus.bowtie2.refB`

- NCBI also allows to download information/annotation about these viruses from their web site. This information has been provided as **Complete_Sequence_info.csv**.

Align the unmapped fastq files to the viral reference

- In this tutorial, we use alignment tool Bowtie2.
- Install Bowtie2 pre-compiled binary file based on instructions in this manual: http://bowtie-bio.sourceforge.net/bowtie2/manual.shtml#obtaining-bowtie-2
- Perform alignment to generate output in the form of a SAM file. This SAM file contains the reads from various viruses aligned to the viral reference.
- Convert SAM to BAM using Samtools. Example command: `samtools view -bS in.sam > out.bam`
- Sort the BAM file coordinate wise. Example command: `samtools sort out.bam > out.sorted`
- Use `Samtools idxstats` tool. Example command: `samtools idxstats out.sorted.bam > idxstats.txt` . This produces a tab delimited file, with each line consisting of a virus sequence name, sequence length, # mapped reads and # unmapped reads. The number of mapped reads is referred to as *genome level counts*. Using the genome level counts, we estimated the number of reads that covered the genome, a form of viral load or viral copy number. Viral copy number was defined as: `the Number of mapped reads * Read Length / Genome Length`

Only those viral species with copy number more than a threshold are selected for the next module.

We have provided this shell script **viral.pipeline_public_final.sh** that encompasses all of these above steps.

Get genome level matrix file and find top viruses

- If you have more than one sample, then it might be useful to concatenate the number of mapped reads (genome level counts) from each sample into a matrix format. This file can then be used for any further analysis.
- We have provided a fully reproducible code in the R programming language in the form of an R markdown file. It contains the code and output for how to merge all the `Samtools idxstats` files. A readable version of this file is here **merge.idx.stats.pdf**. This code also adds virus annotation using information from **Complete_Sequence_info.csv** file.
- Only those viral species with copy number more than a threshold are selected for the next module. For this tutorial, we decided to use a threshold copy number of 10, so this gives us 19 viral genomes used in the next step. This file is available [**here**] (https://docs.google.com/spreadsheets/d/16vSWxLeUdiTXBNzudObbEGFNbXZroWznDnEtjhNF784/edit?usp=sharing) and also shown in the table below .

NC id	Genome Length	Name of virus	SRR1946637 (viGEN read count)	Copy number
NC_018464.1	927	Shamonda virus	82509	8900.65
NC_003977.1	3215	Hepatitis B virus	96329	2996.24
NC_018476.1	6895	Simbu virus	49784	722.03
NC_009823.1	9711	Hepatitis C virus genotype 2	31470	324.07
NC_018478.1	4417	Simbu virus	13745	311.18
NC_018711.1	7195	Lunk virus NKS-1	14251	198.07
NC_004102.1	9646	Hepatitis C virus	17922	185.8
NC_001672.1	11141	Tick-borne encephalitis virus	16336	146.63
NC_015521.1	7310	Cutthroat trout virus	10122	138.47
NC_022518.1	9472	Human endogenous retrovirus K113	9028	95.31

NC id	Genome Length	Name of virus	SRR1946637 (viGEN read count)	Copy number
NC_018382.1	6796	Bat hepevirus	6232	91.7
NC_009827.1	9628	Hepatitis C virus genotype 6	8438	87.64
NC_009225.1	3245	Torque teno midi virus 1	1581	48.72
NC_014093.1	3253	Torque teno midi virus 2	1111	34.15
NC_027202.1	1904	Punta Toro virus	580	30.46
NC_001526.2	7905	Human papillomavirus type 16	1976	25
NC_002645.1	27317	Human coronavirus 229E	5080	18.6
NC_009824.1	9456	Hepatitis C virus genotype 3	1476	15.61
NC_010708.1	3621	Thottapalayam virus	416	11.49

Gene and CDS quantification

- In this step, we use our in-house pipeline to generate gene and CDS counts is for every input BAM file.
- The region level information is extracted from Gene Feature Format files (GFF) files which are available for most viral genomes from NCBI.
- Download GFF files for the top viruses.
- For this tutorial, we have provided the GFF files here **un_bowtie_topGff**. Not all viruses have GFF files. In our example 18 of the 19 viruses had GFF files for download.
- Input to our in-house pipeline for Gene & CDS quantification: the viral bam files (the BAM file output from previous Bowtie2 step)
- We have provided fully reproducible code in the R programming language in the form of an R markdown file **count.regions.in.regions.Rmd** in this github repository, that generates these Gene and CDS counts. A readable version of this file is also provided here: https://github.com/ICBI/viGEN/blob/master/count.reads.in.regions.pdf *We plan to integrate this code into our Bioconductor package.*
- Output :
    - This code produces counts for each region in the GFF file. One file is created for each virus (GFF file) for each sample. Three types of counts are calculated - "in region", "on boundary" and "in gaps". This output is available as a ".csv" file and "Rdata" files.
    - Collate all read counts across all samples and across all top viruses to create a matrix. This code is provided as a fully reproducible code in the R programming language in the form of an R markdown file **collate.output.files.Rmd**. A readable version of this file is also provided here: https://github.com/ICBI/viGEN/blob/master/collate.output.files.pdf. The code calculates total of "in region" and "on boundary" (referred to as "sum") when collating. This code will also add virus annotation using information from "Complete_Sequence_info.csv" file.
    - Application: These gene and CDS count files can be used to compare case and control groups of interest using popular tools like EdgeR (http://bioconductor.org/packages/edgeR/) or DESeq2 in Bioconductor that can accept raw counts.

The viruses that have the highest region counts are shown in table **here**. You can see various gene/CDS regions of Hepatitis B virus showing up on top with highest counts. This is a verification of the HBV status of the sample.

Variant calling

- Install Varscan2 from here: https://sourceforge.net/projects/varscan/files/
- Run Varscan2 on command line using the following command: `samtools mpileup -B -f /Users/ls483/Documents/SRA.GEO/viral.reference/viruses.fa -d 9999 -Q 17 -q 17 SRR1946637_un.bowtie2.sorted.bam|`

```
java -Xmx2g -jar /Users/ls483/Documents/software/varscan2/VarScan.v2.3.9.jar mpileup2cns --output-vcf 1 --min-var-freq 0.01 | awk '/^#/ || $7=="PASS"' > /Users/ls483/Documents/SRA.GEO/output_varscan2/SRR1946637_un.vcf
```

- This produces a the variants found in viruses in a standard variant call file (VCF) file format.

Citation

Please cite our work in BioxRiv available at this link: http://www.biorxiv.org/content/early/2017/01/11/099788). DOI https://doi.org/10.1101/099788.

If you are using these samples for testing this pipeline, please remember to cite this dataset from NCBI

- NCBI SRA http://www.ncbi.nlm.nih.gov/bioproject/PRJNA279878
- EBI SRA http://www.ebi.ac.uk/ena/data/view/SRR1946637

Notes

Rmd files are R Markdown documents that contain narrative text and code in a nicely formatted document, that allow users to completely reproduce the code. Along with the R Markdown files, its PDF version have also been provided for users. The R Markdown files and PDF files have the same name and differ only by the file extension (.Rmd for R Markdown and .pdf for PDF files).
