# Supplementary material for: viGEN: An Open Source Pipeline for the Detection and Quantification of Viral RNA in Human Tumors
Source: Front Microbiol. 2018 Jun 5;9:1172. doi: 10.3389/fmicb.2018.01172 (PMC5996193; doi:10.3389/fmicb.2018.01172)
Supplement: Additional File 2 — Detailed results from analysis of TCGA cervical cancer patients. [file Data_Sheet_2.DOCX]

# Additional File 2

## Estimation of sensitivity and specificity for HPV-16 detection in Cervical Cancer samples

- Consider read count > 4000 to be positive value
- Select those 22 patients for which HPV status was available in clinical data file (performed using PCR or other screening technique)

**Table S2-A: Comparing detection from viGEN with Centrifuge, Kraken and TCGA Clinical Data**

| **case_id** | **viGEN copy number for HPV-16 (K02718.1)** | **Detection of HPV-16 by viGEN** | **Detectionof HPV-16 by Centrifuge*** | **Detectionof HPV-16 by Kraken*** | **Human.papillomavirus.type (From the clinical data)** |
| --- | --- | --- | --- | --- | --- |
| TCGA-C5-A1BF | 0.4 | negative | negative | negative | HPV 18 |
| TCGA-C5-A1BK | 35.0 | positive | positive | negative | HPV 16\|Other HPV type(s) |
| TCGA-C5-A1BL | 65.5 | positive | positive | positive | HPV 16 |
| TCGA-C5-A1M6 | 0.4 | negative | negative | positive | HPV 18 |
| TCGA-C5-A1M7 | 0.0 | negative | negative | positive | HPV 18 |
| TCGA-C5-A1M9 | 433.2 | positive | positive | positive | HPV 16 |
| TCGA-C5-A1ME | 0.1 | negative | negative | negative | HPV 16 |
| TCGA-C5-A1MF | 0.1 | negative | negative | negative | Other HPV type(s) |
| TCGA-C5-A1MK | 0.0 | negative | negative | positive | Other HPV type(s) |
| TCGA-C5-A1ML | 91.4 | positive | positive | positive | HPV 16 |
| TCGA-C5-A2LT | 0.0 | negative | negative | positive | Other HPV type(s) |
| TCGA-C5-A2LV | 87.2 | positive | positive | positive | HPV 16 |
| TCGA-C5-A2LX | 172.4 | positive | positive | positive | HPV 16 |
| TCGA-C5-A2LY | 92.4 | positive | positive | positive | HPV 16 |
| TCGA-C5-A2M1 | 139.3 | positive | positive | positive | HPV 16 |
| TCGA-EK-A2RE | 45.7 | positive | positive | positive | Other HPV type(s) |
| TCGA-EK-A3GK | 104.0 | positive | positive | positive | HPV 16 |
| TCGA-EX-A69M | 0.3 | negative | negative | positive | HPV 16\|Other HPV type(s) |
| TCGA-HM-A3JJ | 35.1 | positive | positive | positive | Other HPV type(s) |
| TCGA-JX-A3Q8 | 265.6 | positive | positive | positive | HPV 16\|HPV 18\|Other HPV type(s) |
| TCGA-MA-AA3Z | 33.2 | positive | positive | positive | Other HPV type(s) |
| TCGA-MU-A51Y | 37.5 | positive | positive | positive | Other HPV type(s) |

*Analysis performed by the Seven Bridges team. Reference: <https://www.sevenbridges.com/centrifuge/>

| viGEN detection of HPV 16 | |  |  | |  |  |  | |  |
| --- | --- | --- | --- | --- | --- | --- | --- | --- | --- |
|  |  |  |  | |  |  |  | |  |
|  |  | PCR or other lab techniques for screening viruses (obtained from clinical data) | | |  |  |  | |  |
|  |  | **Positive** | | **Negative** | **Total** |  |  | |  |
| RNA-seq from tumor tissue (output of algorithmm) | **Positive** | 10 | | 4 | 14 |  |  | |  |
|  | **Negative** | 2 | | 6 | 8 |  | |  | |
|  | **Total** | 12 | | 10 | 22 |  |  | |  |
| Sensitivity= | 83% |  |  |  |  |  |  |  |  |
| Specificity= | 60% |  |  |  |  |  |  |  |  |

| Kraken detection of HPV 16* | |  |  |  |  |
| --- | --- | --- | --- | --- | --- |
|  |  |  |  |  |  |
|  |  | PCR or other lab techniques for screening viruses (obtained from clinical data) | |  |  |
|  |  | **Positive** | **Negative** | **Total** |  |
| RNA-seq from tumor tissue (output of algorithmm) | **Positive** | 10 | 8 | 18 |  |
|  | **Negative** | 2 | 2 | 4 |  |
|  | **Total** | 12 | 10 | 22 |  |
| Sensitivity= | 83% |  |  |  |  |
| Specificity= | 20% |  |  |  |  |
|  |  |  |  |  |  |
| Centrifuge detection of HPV 16* | |  |  |  |  |
|  |  |  |  |  |  |
|  |  | PCR or other lab techniques for screening viruses (obtained from clinical data) | |  |  |
|  |  | **Positive** | **Negative** | **Total** |  |
| RNA-seq from tumor tissue (output of algorithmm) | **Positive** | 10 | 4 | 14 |  |
|  | **Negative** | 2 | 6 | 8 |  |
|  | **Total** | 12 | 10 | 22 |  |
|  |  |  |  |  |  |
| Sensitivity= | 83% |  |  |  |  |
| Specificity= | 60% |  |  |  |  |

## Estimation of sensitivity and specificity for HPV18 detection in Cervical Cancer samples

- Consider read count > 4000 to be positive value
- Select those 22 patients for which HPV status was available in clinical data file (performed using PCR or other screening technique)

**Table S2-B: Comparing detection from viGEN with Centrifuge, Kraken and TCGA Clinical Data**

| case_id | viGEN copy number for HPV-18 (NC_001357.1) | Detection by viGEN | Detectionof HPV-18 by Kraken* | Detectionof HPV-18 by Centrifuge* | Human papillomavirus type (from clinical data) |
| --- | --- | --- | --- | --- | --- |
| TCGA-C5-A1BF | 14.7 | positive | negative | positive | HPV 18 |
| TCGA-C5-A1BK | 0.0 | negative | negative | negative | HPV 16\|Other HPV type(s) |
| TCGA-C5-A1BL | 0.0 | negative | positive | negative | HPV 16 |
| TCGA-C5-A1M6 | 33.0 | positive | positive | positive | HPV 18 |
| TCGA-C5-A1M7 | 54.0 | positive | positive | positive | HPV 18 |
| TCGA-C5-A1M9 | 0.0 | negative | positive | negative | HPV 16 |
| TCGA-C5-A1ME | 16.3 | positive | negative | positive | HPV 16 |
| TCGA-C5-A1MF | 0.0 | negative | negative | negative | Other HPV type(s) |
| TCGA-C5-A1MK | 0.0 | negative | positive | negative | Other HPV type(s) |
| TCGA-C5-A1ML | 0.0 | negative | positive | negative | HPV 16 |
| TCGA-C5-A2LT | 0.0 | negative | positive | negative | Other HPV type(s) |
| TCGA-C5-A2LV | 0.0 | negative | positive | negative | HPV 16 |
| TCGA-C5-A2LX | 0.0 | negative | positive | negative | HPV 16 |
| TCGA-C5-A2LY | 0.0 | negative | positive | negative | HPV 16 |
| TCGA-C5-A2M1 | 0.0 | negative | positive | negative | HPV 16 |
| TCGA-EK-A2RE | 0.0 | negative | positive | negative | Other HPV type(s) |
| TCGA-EK-A3GK | 0.0 | negative | positive | negative | HPV 16 |
| TCGA-EX-A69M | 0.0 | negative | positive | negative | HPV 16\|Other HPV type(s) |
| TCGA-HM-A3JJ | 0.0 | negative | positive | negative | Other HPV type(s) |
| TCGA-JX-A3Q8 | 0.0 | negative | positive | negative | HPV 16\|HPV 18\|Other HPV type(s) |
| TCGA-MA-AA3Z | 0.0 | negative | positive | negative | Other HPV type(s) |
| TCGA-MU-A51Y | 0.0 | negative | positive | negative | Other HPV type(s) |

* Analysis performed by the Seven Bridges team. Reference: <https://www.sevenbridges.com/centrifuge/>

## Comparing viGEN with metagenomic tools Centrifuge and Kraken

We compared the HPV detection ability of viGEN with two metagenomic tools Centrifuge and Kraken. In the graphs below, we compare the fraction of samples where HPV virus was detected.

**Figure S2-A: Most abundant species detected in (a) Centrifuge, (b) Kaken and (c) viGEN**


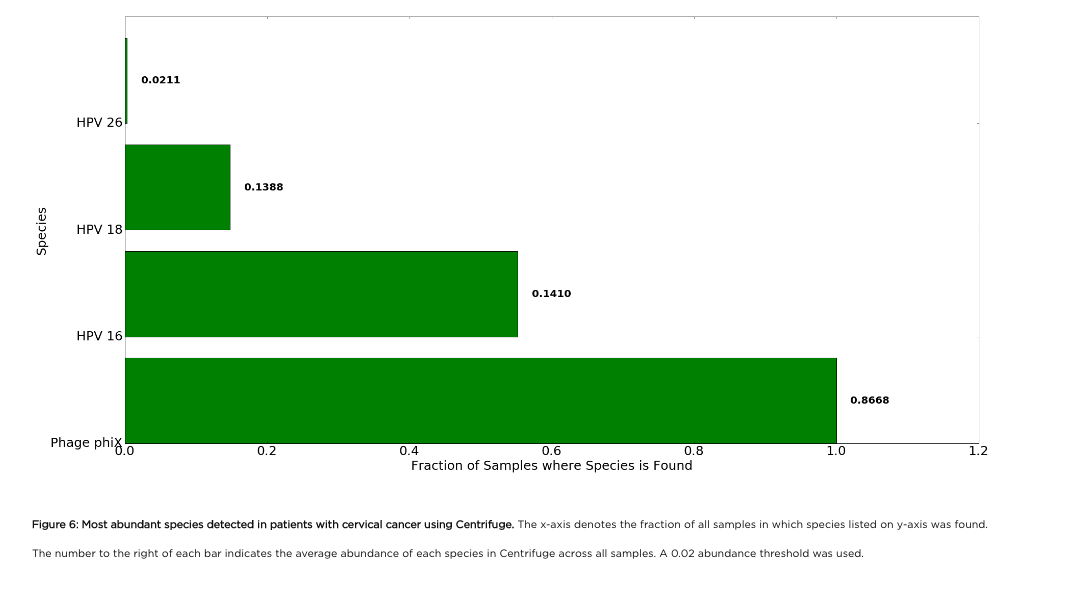


(a) Centrifuge Image taken from: <https://www.sevenbridges.com/centrifuge/>


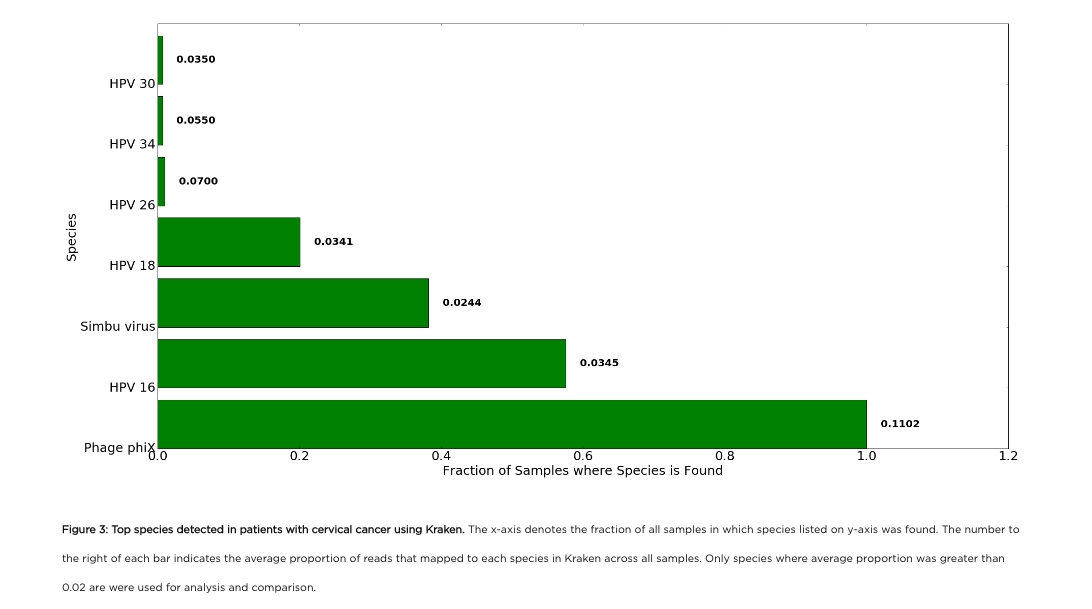


(b) Kraken Image taken from: <https://www.sevenbridges.com/centrifuge/>

(c) viGEN

| **Table S2-C: Summary of species detected in viGEN, Centrifuge and Kraken** | | |
| --- | --- | --- |
| Virus Name | Tool Name | Fraction of samples where the species was found |
| HPV 16 | Centrifuge | 55% |
| HPV 16 (Alphapapillomavirus 9) | Kraken | 58% |
| HPV 16 | viGEN | 53% |
|  |  |  |
| HPV 18 (Alphapapillomavirus 7) | Centrifuge | 15% |
| HPV 18 | Kraken | 20% |
| HPV 18 | viGEN | 13% |
|  |  |  |
| HPV 26 | Centrifuge | 0.3% |
| HPV 26 (Alphapapillomavirus 5) | Kraken | 1% |
| HPV 26 | viGEN | 0.3% |
|  |  |  |
| Phage phiX (control) | Centrifuge | 100% |
| Phage phiX (control) | Kraken | 100% |
| Phage phiX (control) | viGEN | 100% |

**Comparison of viral detection for HPV 26 viGEN, Centrifuge and Kraken**

| **Tool** | **Number of samples detected** | **Sample(s) where the HPV26 virus was detected** |
| --- | --- | --- |
| viGEN | 1/304 (0.3 %) | TCGA-JW-A5VK-01A |
| Centrifuge | 1/304 (0.3 %) | TCGA-JW-A5VK-01A |
| Kraken | 3/304 (1%) | TCGA-JW-A5VK-01A  TCGA-DS-A3LQ-01A  TCGA-VS-A8Q9-01A |

**References:**

- Malhotra et al (2017), Enabling scalable and rapid metagenomic profiling of the transcriptome with the Seven Bridges Cancer Genomics Cloud. Bio IT world. https://www.sevenbridges.com/centrifuge/, https://www.sevenbridges.com/events/bioit-world-2017/
- Kim et al, (2016) Centrifuge: rapid and sensitive classification of metagenomic sequences. Genome Biology
- Wood et al (2014) Kraken: ultrafast metagenomic sequence classification using exact alignments. Genome Biology
