# Supplementary material for: viGEN: An Open Source Pipeline for the Detection and Quantification of Viral RNA in Human Tumors
Source: Front Microbiol. 2018 Jun 5;9:1172. doi: 10.3389/fmicb.2018.01172 (PMC5996193; doi:10.3389/fmicb.2018.01172)
Supplement: Additional File 3 — Output from Kraken and Centrifuge shared by the Seven Bridges Team. [file Data_Sheet_3.DOCX]

# Additional File 3

## Output from Kraken and Centrifuge shared by the Seven Bridges Team

print(kraken_list)

print(centrifuge_list)

TCGA-ZJ-AAXJ-01A

['Enterobacteria phage phiX174 sensu lato', 'Pandoravirus salinus', 'Human endogenous retrovirus K', 'Choristoneura occidentalis granulovirus', 'Alphapapillomavirus 9']

['Enterobacteria phage phiX174 sensu lato', 'Human papillomavirus type 16', 'Human endogenous retrovirus K113', 'Encephalomyocarditis virus', 'Saccharomyces cerevisiae killer virus M1']

(57, 109)

TCGA-EK-A2H0-01A

['Enterobacteria phage phiX174 sensu lato', 'Alphapapillomavirus 7', 'Simbu virus', 'Shamonda virus', 'Choristoneura occidentalis granulovirus']

['Enterobacteria phage phiX174 sensu lato', 'Human endogenous retrovirus K113', 'Saccharomyces cerevisiae killer virus M1', 'Primula malacoides virus China/Mar2007', 'Gentian ovary ring-spot virus']

(285, 281)

TCGA-VS-A958-01A

['Enterobacteria phage phiX174 sensu lato', 'Pandoravirus salinus', 'Human endogenous retrovirus K', 'Tupaiid herpesvirus 1', 'Elephant endotheliotropic herpesvirus 4']

['Enterobacteria phage phiX174 sensu lato', 'Human endogenous retrovirus K113', 'Primula malacoides virus China/Mar2007', 'Saccharomyces cerevisiae killer virus M1', 'Bovine viral diarrhea virus 1']

(121, 29)

TCGA-BI-A20A-01A

['Enterobacteria phage phiX174 sensu lato', 'Alphapapillomavirus 9', 'Simbu virus', 'Human endogenous retrovirus K', 'Shamonda virus']

['Enterobacteria phage phiX174 sensu lato', 'Human papillomavirus type 16', 'Human endogenous retrovirus K113', 'Saccharomyces cerevisiae killer virus M1', 'White clover cryptic virus 2']

(238, 209)

TCGA-C5-A8YR-01A

['Enterobacteria phage phiX174 sensu lato', 'Alphapapillomavirus 7', 'Simbu virus', 'Shamonda virus', 'Choristoneura occidentalis granulovirus']

['Enterobacteria phage phiX174 sensu lato', 'Human endogenous retrovirus K113', 'Primula malacoides virus China/Mar2007', 'Bovine viral diarrhea virus 1', 'Saccharomyces cerevisiae killer virus M1']

(81, 58)

TCGA-C5-A3HL-01A

['Alphapapillomavirus 9', 'Enterobacteria phage phiX174 sensu lato', 'Pandoravirus salinus', 'Simbu virus', 'Human endogenous retrovirus K']

['Enterobacteria phage phiX174 sensu lato', 'Human papillomavirus type 16', 'Human endogenous retrovirus K113', 'Saccharomyces cerevisiae killer virus M1', 'Primula malacoides virus China/Mar2007']

(150, 162)

TCGA-HM-A3JK-01A

['Enterobacteria phage phiX174 sensu lato', 'Alphapapillomavirus 9', 'Simbu virus', 'Shamonda virus', 'Choristoneura occidentalis granulovirus']

['Enterobacteria phage phiX174 sensu lato', 'Human endogenous retrovirus K113', 'Encephalomyocarditis virus', 'Bovine viral diarrhea virus 1', 'Woolly monkey sarcoma virus']

(127, 14)

TCGA-C5-A1BK-01B

['Enterobacteria phage phiX174 sensu lato', 'Simbu virus', 'Shamonda virus', 'Pandoravirus salinus', 'Human endogenous retrovirus K']

['Enterobacteria phage phiX174 sensu lato', 'Human papillomavirus type 16', 'Human endogenous retrovirus K113', 'Saccharomyces cerevisiae killer virus M1', 'Red clover cryptic virus 2']

(284, 282)

TCGA-ZJ-AB0H-01A

['Enterobacteria phage phiX174 sensu lato', 'Alphapapillomavirus 7', 'Human endogenous retrovirus K', 'Pandoravirus salinus', 'Tupaiid herpesvirus 1']

['Enterobacteria phage phiX174 sensu lato', 'Alphapapillomavirus 7', 'Human endogenous retrovirus K113', 'Saccharomyces cerevisiae killer virus M1', 'Bovine viral diarrhea virus 1']

(9, 141)

TCGA-JW-A5VI-01A

['Enterobacteria phage phiX174 sensu lato', 'Alphapapillomavirus 6', 'Pandoravirus salinus', 'Human endogenous retrovirus K', 'Rosellinia necatrix partitivirus 2']

['Enterobacteria phage phiX174 sensu lato', 'Human endogenous retrovirus K113', 'Primula malacoides virus China/Mar2007', 'Red clover cryptic virus 2', 'Bovine viral diarrhea virus 1']

(4, 142)

TCGA-LP-A5U2-01A

['Enterobacteria phage phiX174 sensu lato', 'Alphapapillomavirus 9', 'Human endogenous retrovirus K', 'Elephant endotheliotropic herpesvirus 4', 'Simbu virus']

['Enterobacteria phage phiX174 sensu lato', 'Human papillomavirus type 16', 'Human endogenous retrovirus K113', 'Primula malacoides virus China/Mar2007', 'Saccharomyces cerevisiae killer virus M1']

(161, 156)

TCGA-EA-A78R-01A

['Enterobacteria phage phiX174 sensu lato', 'Pandoravirus salinus', 'Choristoneura occidentalis granulovirus', 'Human endogenous retrovirus K', 'Macacine herpesvirus 1']

['Enterobacteria phage phiX174 sensu lato', 'Human endogenous retrovirus K113', 'Saccharomyces cerevisiae killer virus M1', 'Encephalomyocarditis virus', 'Red clover cryptic virus 2']

(194, 238)

TCGA-VS-A8EB-01A

['Enterobacteria phage phiX174 sensu lato', 'Alphapapillomavirus 9', 'Human endogenous retrovirus K', 'Pandoravirus salinus', 'Simbu virus']

['Enterobacteria phage phiX174 sensu lato', 'Human papillomavirus type 16', 'Human endogenous retrovirus K113', 'Primula malacoides virus China/Mar2007', 'Red clover cryptic virus 2']

(114, 31)

TCGA-C5-A0TN-01A

['Enterobacteria phage phiX174 sensu lato', 'Shamonda virus', 'Simbu virus', 'Choristoneura occidentalis granulovirus', 'Pandoravirus salinus']

['Enterobacteria phage phiX174 sensu lato', 'Human papillomavirus type 16', 'Human endogenous retrovirus K113', 'Saccharomyces cerevisiae killer virus M1', 'Primula malacoides virus China/Mar2007']

(263, 295)

TCGA-ZJ-AAX4-01A

['Enterobacteria phage phiX174 sensu lato', 'Pandoravirus salinus', 'Human endogenous retrovirus K', 'Simbu virus', 'Tupaiid herpesvirus 1']

['Enterobacteria phage phiX174 sensu lato', 'Human papillomavirus type 16', 'Human endogenous retrovirus K113', 'Bovine viral diarrhea virus 1', 'Saccharomyces cerevisiae killer virus M1']

(16, 129)

TCGA-EA-A5ZD-01A

['Enterobacteria phage phiX174 sensu lato', 'Human endogenous retrovirus K', 'Pandoravirus salinus', 'Alphapapillomavirus 9', 'Tupaiid herpesvirus 1']

['Enterobacteria phage phiX174 sensu lato', 'Human endogenous retrovirus K113', 'Saccharomyces cerevisiae killer virus M1', 'Primula malacoides virus China/Mar2007', 'White clover cryptic virus 2']

(199, 232)

TCGA-C5-A7X8-01A

['Enterobacteria phage phiX174 sensu lato', 'Alphapapillomavirus 7', 'Choristoneura occidentalis granulovirus', 'Shamonda virus', 'Human endogenous retrovirus K']

['Enterobacteria phage phiX174 sensu lato', 'Alphapapillomavirus 7', 'Human endogenous retrovirus K113', 'Encephalomyocarditis virus', 'Primula malacoides virus China/Mar2007']

(286, 288)

TCGA-VS-A9UD-01A

['Enterobacteria phage phiX174 sensu lato', 'Alphapapillomavirus 9', 'Human endogenous retrovirus K', 'Pandoravirus salinus', 'Vicia cryptic virus']

['Enterobacteria phage phiX174 sensu lato', 'Human papillomavirus type 16', 'Human endogenous retrovirus K113', 'Saccharomyces cerevisiae killer virus M1', 'Red clover cryptic virus 2']

(101, 41)

TCGA-EK-A2PG-01A

['Alphapapillomavirus 9', 'Enterobacteria phage phiX174 sensu lato', 'Choristoneura occidentalis granulovirus', 'Shamonda virus', 'Pandoravirus salinus']

['Enterobacteria phage phiX174 sensu lato', 'Human endogenous retrovirus K113', 'Saccharomyces cerevisiae killer virus M1', 'Red clover cryptic virus 2', 'Primula malacoides virus China/Mar2007']

(54, 111)

TCGA-EK-A2H1-01A

['Enterobacteria phage phiX174 sensu lato', 'Pandoravirus salinus', 'Simbu virus', 'Shamonda virus', 'Human endogenous retrovirus K']

['Enterobacteria phage phiX174 sensu lato', 'Human papillomavirus type 16', 'Human endogenous retrovirus K113', 'Saccharomyces cerevisiae killer virus M1', 'Primula malacoides virus China/Mar2007']

(8, 139)

TCGA-JW-A5VJ-01A

['Enterobacteria phage phiX174 sensu lato', 'Human endogenous retrovirus K', 'Pandoravirus salinus', 'Alphapapillomavirus 7', 'Tupaiid herpesvirus 1']

['Enterobacteria phage phiX174 sensu lato', 'Alphapapillomavirus 7', 'Human endogenous retrovirus K113', 'Primula malacoides virus China/Mar2007', 'Saccharomyces cerevisiae killer virus M1']

(143, 2)

TCGA-BI-A0VR-01A

['Enterobacteria phage phiX174 sensu lato', 'Simbu virus', 'Shamonda virus', 'Pandoravirus salinus', 'Choristoneura occidentalis granulovirus']

['Enterobacteria phage phiX174 sensu lato', 'Human papillomavirus type 16', 'Human endogenous retrovirus K113', 'Saccharomyces cerevisiae killer virus M1', 'Primula malacoides virus China/Mar2007']

(123, 35)

TCGA-C5-A1MI-01A

['Enterobacteria phage phiX174 sensu lato', 'Alphapapillomavirus 7', 'Human endogenous retrovirus K', 'Simbu virus', 'Pandoravirus salinus']

['Enterobacteria phage phiX174 sensu lato', 'Alphapapillomavirus 7', 'Human endogenous retrovirus K113', 'Saccharomyces cerevisiae killer virus M1', 'White clover cryptic virus 2']

(245, 202)

TCGA-VS-A8QH-01A

['Enterobacteria phage phiX174 sensu lato', 'Simbu virus', 'Shamonda virus', 'Choristoneura occidentalis granulovirus', 'Macacine herpesvirus 1']

['Enterobacteria phage phiX174 sensu lato', 'Human endogenous retrovirus K113', 'Encephalomyocarditis virus', 'Primula malacoides virus China/Mar2007', 'Red clover cryptic virus 2']

(282, 285)

TCGA-VS-A9UH-01A

['Enterobacteria phage phiX174 sensu lato', 'Pandoravirus salinus', 'Human endogenous retrovirus K', 'Rosellinia necatrix partitivirus 2', 'Tupaiid herpesvirus 1']

['Enterobacteria phage phiX174 sensu lato', 'Human endogenous retrovirus K113', 'Primula malacoides virus China/Mar2007', 'Saccharomyces cerevisiae killer virus M1', 'White clover cryptic virus 2']

(266, 297)

TCGA-ZJ-AAXF-01A

['Enterobacteria phage phiX174 sensu lato', 'Pandoravirus salinus', 'Human endogenous retrovirus K', 'Choristoneura occidentalis granulovirus', 'Alphapapillomavirus 9']

['Enterobacteria phage phiX174 sensu lato', 'Human papillomavirus type 16', 'Human endogenous retrovirus K113', 'Saccharomyces cerevisiae killer virus M1', 'Bovine viral diarrhea virus 1']

(190, 191)

TCGA-JW-A852-01A

['Enterobacteria phage phiX174 sensu lato', 'Pandoravirus salinus', 'Human endogenous retrovirus K', 'Choristoneura occidentalis granulovirus', 'Shamonda virus']

['Enterobacteria phage phiX174 sensu lato', 'Human endogenous retrovirus K113', 'Encephalomyocarditis virus', 'Human papillomavirus type 16', 'White clover cryptic virus 2']

(251, 223)

TCGA-VS-A94W-01A

['Enterobacteria phage phiX174 sensu lato', 'Alphapapillomavirus 9', 'Macacine herpesvirus 1', 'Human endogenous retrovirus K', 'Pandoravirus salinus']

['Enterobacteria phage phiX174 sensu lato', 'Human papillomavirus type 16', 'Human endogenous retrovirus K113', 'Encephalomyocarditis virus', 'Bovine viral diarrhea virus 1']

(86, 60)

TCGA-IR-A3LL-01A

['Enterobacteria phage phiX174 sensu lato', 'Human endogenous retrovirus K', 'Pandoravirus salinus', 'Alphapapillomavirus 9', 'Rosellinia necatrix partitivirus 2']

['Enterobacteria phage phiX174 sensu lato', 'Human papillomavirus type 16', 'Primula malacoides virus China/Mar2007', 'Human endogenous retrovirus K113', 'Red clover cryptic virus 2']

(281, 283)

TCGA-ZJ-AB0I-01A

['Enterobacteria phage phiX174 sensu lato', 'Pandoravirus salinus', 'Alphapapillomavirus 9', 'Human endogenous retrovirus K', 'Pandoravirus dulcis']

['Enterobacteria phage phiX174 sensu lato', 'Human papillomavirus type 16', 'Human endogenous retrovirus K113', 'Saccharomyces cerevisiae killer virus M1', 'Primula malacoides virus China/Mar2007']

(23, 123)

TCGA-EA-A556-01A

['Enterobacteria phage phiX174 sensu lato', 'Human endogenous retrovirus K', 'Simbu virus', 'Shamonda virus', 'Pandoravirus salinus']

['Enterobacteria phage phiX174 sensu lato', 'Human endogenous retrovirus K113', 'White clover cryptic virus 2', 'Saccharomyces cerevisiae killer virus M1', 'Hepatitis C virus']

(160, 152)

TCGA-EX-A69L-01A

['Enterobacteria phage phiX174 sensu lato', 'Alphapapillomavirus 9', 'Human endogenous retrovirus K', 'Pandoravirus salinus', 'Vicia cryptic virus']

['Enterobacteria phage phiX174 sensu lato', 'Human papillomavirus type 16', 'Human endogenous retrovirus K113', 'White clover cryptic virus 2', 'Primula malacoides virus China/Mar2007']

(267, 300)

TCGA-VS-A9V3-01A

['Enterobacteria phage phiX174 sensu lato', 'Alphapapillomavirus 9', 'Human endogenous retrovirus K', 'Pandoravirus salinus', 'Tupaiid herpesvirus 1']

['Enterobacteria phage phiX174 sensu lato', 'Human endogenous retrovirus K113', 'Y73 sarcoma virus', 'Bovine viral diarrhea virus 1', 'Encephalomyocarditis virus']

(15, 131)

TCGA-EA-A5FO-01A

['Enterobacteria phage phiX174 sensu lato', 'Alphapapillomavirus 9', 'Human endogenous retrovirus K', 'Pandoravirus salinus', 'Choristoneura occidentalis granulovirus']

['Enterobacteria phage phiX174 sensu lato', 'Human papillomavirus type 16', 'Human endogenous retrovirus K113', 'Primula malacoides virus China/Mar2007', 'White clover cryptic virus 2']

(171, 170)

TCGA-MU-A5YI-01A

['Enterobacteria phage phiX174 sensu lato', 'Choristoneura occidentalis granulovirus', 'Human endogenous retrovirus K', 'Pandoravirus salinus', 'Shamonda virus']

['Enterobacteria phage phiX174 sensu lato', 'Human papillomavirus type 16', 'Human endogenous retrovirus K113', 'Saccharomyces cerevisiae killer virus M1', 'Primula malacoides virus China/Mar2007']

(169, 165)

TCGA-Q1-A6DV-01A

['Alphapapillomavirus 9', 'Enterobacteria phage phiX174 sensu lato', 'Pandoravirus salinus', 'Human endogenous retrovirus K', 'Bacillus virus SPO1']

['Enterobacteria phage phiX174 sensu lato', 'Human papillomavirus type 16', 'Human endogenous retrovirus K113', 'Saccharomyces cerevisiae killer virus M1', 'Primula malacoides virus China/Mar2007']

(191, 190)

TCGA-C5-A7X3-01A

['Enterobacteria phage phiX174 sensu lato', 'Alphapapillomavirus 7', 'Simbu virus', 'Shamonda virus', 'Choristoneura occidentalis granulovirus']

['Enterobacteria phage phiX174 sensu lato', 'Human endogenous retrovirus K113', 'Encephalomyocarditis virus', 'Alphapapillomavirus 7', 'Saccharomyces cerevisiae killer virus M1']

(164, 158)

TCGA-C5-A7CK-01A

['Enterobacteria phage phiX174 sensu lato', 'Alphapapillomavirus 9', 'Human endogenous retrovirus K', 'Pandoravirus salinus', 'Pandoravirus dulcis']

['Enterobacteria phage phiX174 sensu lato', 'Human papillomavirus type 16', 'Human endogenous retrovirus K113', 'Primula malacoides virus China/Mar2007', 'Bovine viral diarrhea virus 1']

(88, 53)

TCGA-EA-A3HU-01A

['Enterobacteria phage phiX174 sensu lato', 'Alphapapillomavirus 9', 'Pandoravirus salinus', 'Human endogenous retrovirus K', 'Shamonda virus']

['Enterobacteria phage phiX174 sensu lato', 'Human papillomavirus type 16', 'Human endogenous retrovirus K113', 'Saccharomyces cerevisiae killer virus M1', 'Primula malacoides virus China/Mar2007']

(104, 51)

TCGA-EK-A2PI-01A

['Enterobacteria phage phiX174 sensu lato', 'Alphapapillomavirus 9', 'Pandoravirus salinus', 'Human endogenous retrovirus K', 'Choristoneura occidentalis granulovirus']

['Enterobacteria phage phiX174 sensu lato', 'Human papillomavirus type 16', 'Human endogenous retrovirus K113', 'Red clover cryptic virus 2', 'Saccharomyces cerevisiae killer virus M1']

(158, 153)

TCGA-JW-A69B-01A

['Enterobacteria phage phiX174 sensu lato', 'Alphapapillomavirus 9', 'Human endogenous retrovirus K', 'Pandoravirus salinus', 'Vicia cryptic virus']

['Enterobacteria phage phiX174 sensu lato', 'Human papillomavirus type 16', 'Human endogenous retrovirus K113', 'Encephalomyocarditis virus', 'Saccharomyces cerevisiae killer virus M1']

(66, 77)

TCGA-FU-A3HY-01A

['Enterobacteria phage phiX174 sensu lato', 'Alphapapillomavirus 9', 'Pandoravirus salinus', 'Human endogenous retrovirus K', 'Shamonda virus']

['Enterobacteria phage phiX174 sensu lato', 'Human papillomavirus type 16', 'Human endogenous retrovirus K113', 'Saccharomyces cerevisiae killer virus M1', 'Primula malacoides virus China/Mar2007']

(296, 257)

TCGA-UC-A7PI-01A

['Enterobacteria phage phiX174 sensu lato', 'Alphapapillomavirus 7', 'Human endogenous retrovirus K', 'Macacine herpesvirus 1', 'Pandoravirus salinus']

['Enterobacteria phage phiX174 sensu lato', 'Alphapapillomavirus 7', 'Human endogenous retrovirus K113', 'Encephalomyocarditis virus', 'Bovine viral diarrhea virus 1']

(67, 73)

TCGA-FU-A3YQ-01A

['Enterobacteria phage phiX174 sensu lato', 'Alphapapillomavirus 9', 'Human endogenous retrovirus K', 'Pandoravirus salinus', 'Bacillus virus SPO1']

['Enterobacteria phage phiX174 sensu lato', 'Human papillomavirus type 16', 'Primula malacoides virus China/Mar2007', 'Human endogenous retrovirus K113', 'Saccharomyces cerevisiae killer virus M1']

(73, 68)

TCGA-EK-A3GM-01A

['Enterobacteria phage phiX174 sensu lato', 'Alphapapillomavirus 7', 'Human endogenous retrovirus K', 'Pandoravirus salinus', 'Bacillus virus SPO1']

['Enterobacteria phage phiX174 sensu lato', 'Human endogenous retrovirus K113', 'Saccharomyces cerevisiae killer virus M1', 'White clover cryptic virus 2', 'Primula malacoides virus China/Mar2007']

(49, 91)

TCGA-EX-A1H6-01B

['Enterobacteria phage phiX174 sensu lato', 'Simbu virus', 'Human endogenous retrovirus K', 'Pandoravirus salinus', 'Shamonda virus']

['Enterobacteria phage phiX174 sensu lato', 'Human endogenous retrovirus K113', 'White clover cryptic virus 2', 'Human papillomavirus type 16', 'Saccharomyces cerevisiae killer virus M1']

(255, 274)

TCGA-ZJ-AAX8-01A

['Enterobacteria phage phiX174 sensu lato', 'Alphapapillomavirus 7', 'Pandoravirus salinus', 'Human endogenous retrovirus K', 'Shamonda virus']

['Enterobacteria phage phiX174 sensu lato', 'Human endogenous retrovirus K113', 'Bovine viral diarrhea virus 1', 'Saccharomyces cerevisiae killer virus M1', 'Primula malacoides virus China/Mar2007']

(44, 97)

TCGA-IR-A3LF-01A

['Alphapapillomavirus 9', 'Enterobacteria phage phiX174 sensu lato', 'Human endogenous retrovirus K', 'Pandoravirus salinus', 'Bacillus virus SPO1']

['Enterobacteria phage phiX174 sensu lato', 'Human papillomavirus type 16', 'Human endogenous retrovirus K113', 'Saccharomyces cerevisiae killer virus M1', 'White clover cryptic virus 2']

(178, 184)

TCGA-EA-A6QX-01A

['Enterobacteria phage phiX174 sensu lato', 'Alphapapillomavirus 9', 'Human endogenous retrovirus K', 'Pandoravirus salinus', 'Shamonda virus']

['Enterobacteria phage phiX174 sensu lato', 'Human endogenous retrovirus K113', 'Bovine viral diarrhea virus 1', 'Encephalomyocarditis virus', 'Y73 sarcoma virus']

(25, 121)

TCGA-EA-A4BA-01A

['Enterobacteria phage phiX174 sensu lato', 'Simbu virus', 'Choristoneura occidentalis granulovirus', 'Shamonda virus', 'Human endogenous retrovirus K']

['Enterobacteria phage phiX174 sensu lato', 'White clover cryptic virus 2', 'Human endogenous retrovirus K113', 'Saccharomyces cerevisiae killer virus M1', 'Hepatitis C virus']

(124, 0)

TCGA-DS-A0VK-01A

['Enterobacteria phage phiX174 sensu lato', 'Human endogenous retrovirus K', 'Alphapapillomavirus 9', 'Pandoravirus salinus', 'Simbu virus']

['Enterobacteria phage phiX174 sensu lato', 'Human papillomavirus type 16', 'Human endogenous retrovirus K113', 'Primula malacoides virus China/Mar2007', 'Saccharomyces cerevisiae killer virus M1']

(45, 112)

TCGA-DG-A2KM-01A

['Enterobacteria phage phiX174 sensu lato', 'Pandoravirus salinus', 'Simbu virus', 'Human endogenous retrovirus K', 'Shamonda virus']

['Enterobacteria phage phiX174 sensu lato', 'Human papillomavirus type 16', 'Human endogenous retrovirus K113', 'Saccharomyces cerevisiae killer virus M1', 'Primula malacoides virus China/Mar2007']

(105, 38)

TCGA-C5-A1M8-01A

['Enterobacteria phage phiX174 sensu lato', 'Simbu virus', 'Pandoravirus salinus', 'Shamonda virus', 'Human endogenous retrovirus K']

['Enterobacteria phage phiX174 sensu lato', 'Human endogenous retrovirus K113', 'Human papillomavirus type 16', 'Primula malacoides virus China/Mar2007', 'Red clover cryptic virus 2']

(152, 161)

TCGA-EA-A410-01A

['Enterobacteria phage phiX174 sensu lato', 'Pandoravirus salinus', 'Shamonda virus', 'Choristoneura occidentalis granulovirus', 'Simbu virus']

['Enterobacteria phage phiX174 sensu lato', 'Human endogenous retrovirus K113', 'Bovine viral diarrhea virus 1', 'Encephalomyocarditis virus', 'White clover cryptic virus 2']

(87, 54)

TCGA-C5-A2LZ-01A

['Alphapapillomavirus 9', 'Enterobacteria phage phiX174 sensu lato', 'Human endogenous retrovirus K', 'Pandoravirus salinus', 'Rosellinia necatrix partitivirus 2']

['Enterobacteria phage phiX174 sensu lato', 'Human papillomavirus type 16', 'Human endogenous retrovirus K113', 'Primula malacoides virus China/Mar2007', 'Saccharomyces cerevisiae killer virus M1']

(237, 216)

TCGA-C5-A8YT-01A

['Enterobacteria phage phiX174 sensu lato', 'Simbu virus', 'Shamonda virus', 'Choristoneura occidentalis granulovirus', 'Human endogenous retrovirus K']

['Enterobacteria phage phiX174 sensu lato', 'Human endogenous retrovirus K113', 'Bovine viral diarrhea virus 1', 'Saccharomyces cerevisiae killer virus M1', 'Ball python nidovirus']

(48, 101)

TCGA-IR-A3LA-01A

['Enterobacteria phage phiX174 sensu lato', 'Human endogenous retrovirus K', 'Simbu virus', 'Pandoravirus salinus', 'Bacillus virus SPO1']

['Enterobacteria phage phiX174 sensu lato', 'Human endogenous retrovirus K113', 'White clover cryptic virus 2', 'Saccharomyces cerevisiae killer virus M1', 'Primula malacoides virus China/Mar2007']

(283, 290)

TCGA-VS-A959-01A

['Enterobacteria phage phiX174 sensu lato', 'Alphapapillomavirus 9', 'Human endogenous retrovirus K', 'Pandoravirus salinus', 'Choristoneura occidentalis granulovirus']

['Enterobacteria phage phiX174 sensu lato', 'Human endogenous retrovirus K113', 'Primula malacoides virus China/Mar2007', 'Bovine viral diarrhea virus 1', 'Woolly monkey sarcoma virus']

(176, 180)

TCGA-Q1-A73S-01A

['Enterobacteria phage phiX174 sensu lato', 'Simbu virus', 'Shamonda virus', 'Choristoneura occidentalis granulovirus', 'Alphapapillomavirus 7']

['Enterobacteria phage phiX174 sensu lato', 'Alphapapillomavirus 7', 'Human endogenous retrovirus K113', 'Encephalomyocarditis virus', 'Primula malacoides virus China/Mar2007']

(27, 119)

TCGA-MU-A51Y-01A

['Enterobacteria phage phiX174 sensu lato', 'Simbu virus', 'Alphapapillomavirus 9', 'Pandoravirus salinus', 'Human endogenous retrovirus K']

['Enterobacteria phage phiX174 sensu lato', 'Human papillomavirus type 16', 'Human endogenous retrovirus K113', 'Primula malacoides virus China/Mar2007', 'Bovine viral diarrhea virus 1']

(107, 52)

TCGA-EK-A2R7-01A

['Enterobacteria phage phiX174 sensu lato', 'Human endogenous retrovirus K', 'Alphapapillomavirus 7', 'Simbu virus', 'Pandoravirus salinus']

['Enterobacteria phage phiX174 sensu lato', 'Alphapapillomavirus 7', 'Human endogenous retrovirus K113', 'Primula malacoides virus China/Mar2007', 'Saccharomyces cerevisiae killer virus M1']

(226, 224)

TCGA-VS-A8QC-01A

['Enterobacteria phage phiX174 sensu lato', 'Pandoravirus salinus', 'Macacine herpesvirus 1', 'Human endogenous retrovirus K', 'Alphapapillomavirus 9']

['Enterobacteria phage phiX174 sensu lato', 'Human papillomavirus type 16', 'Human endogenous retrovirus K113', 'Encephalomyocarditis virus', 'Primula malacoides virus China/Mar2007']

(111, 18)

TCGA-C5-A1BE-01B

['Enterobacteria phage phiX174 sensu lato', 'Simbu virus', 'Shamonda virus', 'Pandoravirus salinus', 'Human endogenous retrovirus K']

['Enterobacteria phage phiX174 sensu lato', 'Human papillomavirus type 16', 'Human endogenous retrovirus K113', 'Saccharomyces cerevisiae killer virus M1', 'Primula malacoides virus China/Mar2007']

(195, 239)

TCGA-PN-A8MA-01A

['Enterobacteria phage phiX174 sensu lato', 'Human endogenous retrovirus K', 'Shamonda virus', 'Choristoneura occidentalis granulovirus', 'Alphapapillomavirus 9']

['Enterobacteria phage phiX174 sensu lato', 'Human papillomavirus type 16', 'Human endogenous retrovirus K113', 'Primula malacoides virus China/Mar2007', 'Saccharomyces cerevisiae killer virus M1']

(96, 43)

TCGA-EK-A2RE-01A

['Enterobacteria phage phiX174 sensu lato', 'Pandoravirus salinus', 'Shamonda virus', 'Human endogenous retrovirus K', 'Alphapapillomavirus 9']

['Enterobacteria phage phiX174 sensu lato', 'Human papillomavirus type 16', 'Human endogenous retrovirus K113', 'Red clover cryptic virus 2', 'Primula malacoides virus China/Mar2007']

(249, 201)

TCGA-VS-A8EI-01A

['Enterobacteria phage phiX174 sensu lato', 'Simbu virus', 'Shamonda virus', 'Human endogenous retrovirus K', 'Choristoneura occidentalis granulovirus']

['Enterobacteria phage phiX174 sensu lato', 'Human papillomavirus type 16', 'Human endogenous retrovirus K113', 'Encephalomyocarditis virus', 'Primula malacoides virus China/Mar2007']

(141, 36)

TCGA-C5-A1BN-01B

['Enterobacteria phage phiX174 sensu lato', 'Simbu virus', 'Choristoneura occidentalis granulovirus', 'Shamonda virus', 'Alphapapillomavirus 7']

['Enterobacteria phage phiX174 sensu lato', 'Human endogenous retrovirus K113', 'White clover cryptic virus 2', 'Saccharomyces cerevisiae killer virus M1', 'Primula malacoides virus China/Mar2007']

(254, 277)

TCGA-VS-A9UZ-01A

['Enterobacteria phage phiX174 sensu lato', 'Alphapapillomavirus 9', 'Human endogenous retrovirus K', 'Pandoravirus salinus', 'Bacillus virus SPO1']

['Enterobacteria phage phiX174 sensu lato', 'Human papillomavirus type 16', 'Human endogenous retrovirus K113', 'Bovine viral diarrhea virus 1', 'Saccharomyces cerevisiae killer virus M1']

(279, 276)

TCGA-VS-A9UB-01A

['Enterobacteria phage phiX174 sensu lato', 'Alphapapillomavirus 7', 'Human endogenous retrovirus K', 'Pandoravirus salinus', 'Rosellinia necatrix partitivirus 2']

['Enterobacteria phage phiX174 sensu lato', 'Human endogenous retrovirus K113', 'Bovine viral diarrhea virus 1', 'Saccharomyces cerevisiae killer virus M1', 'White clover cryptic virus 2']

(209, 249)

TCGA-EA-A97N-01A

['Enterobacteria phage phiX174 sensu lato', 'Alphapapillomavirus 9', 'Human endogenous retrovirus K', 'Pandoravirus salinus', 'Bacillus virus SPO1']

['Enterobacteria phage phiX174 sensu lato', 'Human papillomavirus type 16', 'Human endogenous retrovirus K113', 'Primula malacoides virus China/Mar2007', 'Red clover cryptic virus 2']

(82, 56)

TCGA-C5-A3HF-01A

['Alphapapillomavirus 7', 'Enterobacteria phage phiX174 sensu lato', 'Human endogenous retrovirus K', 'Pandoravirus salinus', 'Alphapapillomavirus 9']

['Enterobacteria phage phiX174 sensu lato', 'Alphapapillomavirus 7', 'Human endogenous retrovirus K113', 'Saccharomyces cerevisiae killer virus M1', 'Gentian ovary ring-spot virus']

(300, 34)

TCGA-DS-A1O9-01A

['Enterobacteria phage phiX174 sensu lato', 'Simbu virus', 'Alphapapillomavirus 11', 'Shamonda virus', 'Choristoneura occidentalis granulovirus']

['Enterobacteria phage phiX174 sensu lato', 'Human endogenous retrovirus K113', 'Saccharomyces cerevisiae killer virus M1', 'Primula malacoides virus China/Mar2007', 'White clover cryptic virus 2']

(128, 5)

TCGA-C5-A1M5-01A

['Alphapapillomavirus 9', 'Enterobacteria phage phiX174 sensu lato', 'Simbu virus', 'Pandoravirus salinus', 'Shamonda virus']

['Enterobacteria phage phiX174 sensu lato', 'Human endogenous retrovirus K113', 'Primula malacoides virus China/Mar2007', 'Saccharomyces cerevisiae killer virus M1', 'Red clover cryptic virus 2']

(173, 187)

TCGA-LP-A7HU-01A

['Enterobacteria phage phiX174 sensu lato', 'Simbu virus', 'Shamonda virus', 'Choristoneura occidentalis granulovirus', 'Alphapapillomavirus 9']

['Enterobacteria phage phiX174 sensu lato', 'Human papillomavirus type 16', 'Human endogenous retrovirus K113', 'Encephalomyocarditis virus', 'Bovine viral diarrhea virus 1']

(20, 125)

TCGA-MY-A5BE-01A

['Simbu virus', 'Enterobacteria phage phiX174 sensu lato', 'Shamonda virus', 'Choristoneura occidentalis granulovirus', 'Human endogenous retrovirus K']

['Enterobacteria phage phiX174 sensu lato', 'Human papillomavirus type 16', 'Human endogenous retrovirus K113', 'White clover cryptic virus 2', 'Saccharomyces cerevisiae killer virus M1']

(78, 61)

TCGA-IR-A3L7-01A

['Enterobacteria phage phiX174 sensu lato', 'Alphapapillomavirus 7', 'Human endogenous retrovirus K', 'Pandoravirus salinus', 'Gentian ovary ring-spot virus']

['Enterobacteria phage phiX174 sensu lato', 'Alphapapillomavirus 7', 'Human endogenous retrovirus K113', 'Gentian ovary ring-spot virus', 'Saccharomyces cerevisiae killer virus M1']

(22, 120)

TCGA-FU-A57G-01A

['Enterobacteria phage phiX174 sensu lato', 'Human endogenous retrovirus K', 'Simbu virus', 'Pandoravirus salinus', 'Rosellinia necatrix partitivirus 2']

['Enterobacteria phage phiX174 sensu lato', 'Human endogenous retrovirus K113', 'White clover cryptic virus 2', 'Woolly monkey sarcoma virus', 'Primula malacoides virus China/Mar2007']

(236, 219)

TCGA-C5-A905-01A

['Enterobacteria phage phiX174 sensu lato', 'Alphapapillomavirus 9', 'Human endogenous retrovirus K', 'Pandoravirus salinus', 'Shamonda virus']

['Enterobacteria phage phiX174 sensu lato', 'Human papillomavirus type 16', 'Human endogenous retrovirus K113', 'Bovine viral diarrhea virus 1', 'Encephalomyocarditis virus']

(42, 106)

TCGA-EK-A2IR-01A

['Enterobacteria phage phiX174 sensu lato', 'Alphapapillomavirus 9', 'Human endogenous retrovirus K', 'Simbu virus', 'Shamonda virus']

['Enterobacteria phage phiX174 sensu lato', 'Human papillomavirus type 16', 'Human endogenous retrovirus K113', 'Saccharomyces cerevisiae killer virus M1', 'Gentian ovary ring-spot virus']

(154, 147)

TCGA-LP-A4AX-01A

['Enterobacteria phage phiX174 sensu lato', 'Alphapapillomavirus 9', 'Human endogenous retrovirus K', 'Pandoravirus salinus', 'Rosellinia necatrix partitivirus 2']

['Enterobacteria phage phiX174 sensu lato', 'Human papillomavirus type 16', 'Human endogenous retrovirus K113', 'Saccharomyces cerevisiae killer virus M1', 'Primula malacoides virus China/Mar2007']

(229, 208)

TCGA-VS-A8EL-01A

['Enterobacteria phage phiX174 sensu lato', 'Alphapapillomavirus 9', 'Pandoravirus salinus', 'Human endogenous retrovirus K', 'Shamonda virus']

['Enterobacteria phage phiX174 sensu lato', 'Human endogenous retrovirus K113', 'Bovine viral diarrhea virus 1', 'Encephalomyocarditis virus', 'Saccharomyces cerevisiae killer virus M1']

(65, 71)

TCGA-HM-A3JJ-01A

['Enterobacteria phage phiX174 sensu lato', 'Pandoravirus salinus', 'Human endogenous retrovirus K', 'Alphapapillomavirus 9', 'Vicia cryptic virus']

['Enterobacteria phage phiX174 sensu lato', 'Human papillomavirus type 16', 'Human endogenous retrovirus K113', 'Saccharomyces cerevisiae killer virus M1', 'White clover cryptic virus 2']

(204, 175)

TCGA-EX-A3L1-01A

['Enterobacteria phage phiX174 sensu lato', 'Alphapapillomavirus 7', 'Choristoneura occidentalis granulovirus', 'Shamonda virus', 'Pandoravirus salinus']

['Enterobacteria phage phiX174 sensu lato', 'Human endogenous retrovirus K113', 'Woolly monkey sarcoma virus', 'Bovine viral diarrhea virus 1', 'Primula malacoides virus China/Mar2007']

(26, 124)

TCGA-EA-A44S-01A

['Enterobacteria phage phiX174 sensu lato', 'Human endogenous retrovirus K', 'Pandoravirus salinus', 'Hepatitis C virus', 'Rosellinia necatrix partitivirus 2']

['Enterobacteria phage phiX174 sensu lato', 'Human endogenous retrovirus K113', 'White clover cryptic virus 2', 'Human papillomavirus type 16', 'Hepatitis C virus']

(155, 150)

TCGA-DG-A2KK-01A

['Enterobacteria phage phiX174 sensu lato', 'Alphapapillomavirus 9', 'Simbu virus', 'Shamonda virus', 'Choristoneura occidentalis granulovirus']

['Enterobacteria phage phiX174 sensu lato', 'Human papillomavirus type 16', 'Human endogenous retrovirus K113', 'Ball python nidovirus', 'Bovine viral diarrhea virus 1']

(268, 301)

TCGA-VS-A9UT-01A

['Enterobacteria phage phiX174 sensu lato', 'Human endogenous retrovirus K', 'Pandoravirus salinus', 'Simbu virus', 'Shamonda virus']

['Enterobacteria phage phiX174 sensu lato', 'Human endogenous retrovirus K113', 'Bovine viral diarrhea virus 1', 'Encephalomyocarditis virus', 'Y73 sarcoma virus']

(80, 65)

TCGA-FU-A3HZ-01A

['Enterobacteria phage phiX174 sensu lato', 'Pandoravirus salinus', 'Volepox virus', 'White spot syndrome virus', 'Human endogenous retrovirus K']

['Enterobacteria phage phiX174 sensu lato', 'Human endogenous retrovirus K113', 'Primula malacoides virus China/Mar2007', 'Saccharomyces cerevisiae killer virus M1', 'Red clover cryptic virus 2']

(206, 250)

TCGA-VS-A94Y-01A

['Enterobacteria phage phiX174 sensu lato', 'Human endogenous retrovirus K', 'Alphapapillomavirus 7', 'Pandoravirus salinus', 'Tupaiid herpesvirus 1']

['Enterobacteria phage phiX174 sensu lato', 'Human endogenous retrovirus K113', 'Primula malacoides virus China/Mar2007', 'Red clover cryptic virus 2', 'Encephalomyocarditis virus']

(62, 83)

TCGA-DG-A2KH-01A

['Enterobacteria phage phiX174 sensu lato', 'Alphapapillomavirus 7', 'Human endogenous retrovirus K', 'Rosellinia necatrix partitivirus 2', 'Gentian ovary ring-spot virus']

['Enterobacteria phage phiX174 sensu lato', 'Alphapapillomavirus 7', 'Primula malacoides virus China/Mar2007', 'Red clover cryptic virus 2', 'Human endogenous retrovirus K113']

(233, 218)

TCGA-EX-A8YF-01A

['Enterobacteria phage phiX174 sensu lato', 'Human endogenous retrovirus K', 'Alphapapillomavirus 7', 'Shamonda virus', 'Choristoneura occidentalis granulovirus']

['Enterobacteria phage phiX174 sensu lato', 'Alphapapillomavirus 7', 'Human endogenous retrovirus K113', 'Encephalomyocarditis virus', 'Bovine viral diarrhea virus 1']

(186, 196)

TCGA-DS-A7WH-01A

['Enterobacteria phage phiX174 sensu lato', 'Alphapapillomavirus 9', 'Macacine herpesvirus 1', 'Cardiovirus A', 'Pandoravirus salinus']

['Enterobacteria phage phiX174 sensu lato', 'Human papillomavirus type 16', 'Encephalomyocarditis virus', 'Human endogenous retrovirus K113', 'Saccharomyces cerevisiae killer virus M1']

(31, 117)

TCGA-EA-A439-01A

['Enterobacteria phage phiX174 sensu lato', 'Alphapapillomavirus 7', 'Human endogenous retrovirus K', 'Pandoravirus salinus', 'Bacillus virus SPO1']

['Enterobacteria phage phiX174 sensu lato', 'Alphapapillomavirus 7', 'Human endogenous retrovirus K113', 'Saccharomyces cerevisiae killer virus M1', 'Primula malacoides virus China/Mar2007']

(294, 266)

TCGA-VS-A9UL-01A

['Enterobacteria phage phiX174 sensu lato', 'Alphapapillomavirus 7', 'Human endogenous retrovirus K', 'Pandoravirus salinus', 'Pandoravirus dulcis']

['Enterobacteria phage phiX174 sensu lato', 'Alphapapillomavirus 7', 'Human endogenous retrovirus K113', 'Saccharomyces cerevisiae killer virus M1', 'Bovine viral diarrhea virus 1']

(213, 251)

TCGA-JW-AAVH-01A

['Enterobacteria phage phiX174 sensu lato', 'Alphapapillomavirus 9', 'Human endogenous retrovirus K', 'Pandoravirus salinus', 'Shamonda virus']

['Enterobacteria phage phiX174 sensu lato', 'Human papillomavirus type 16', 'Human endogenous retrovirus K113', 'Primula malacoides virus China/Mar2007', 'Saccharomyces cerevisiae killer virus M1']

(2, 143)

TCGA-Q1-A5R2-01A

['Enterobacteria phage phiX174 sensu lato', 'Alphapapillomavirus 9', 'Human endogenous retrovirus K', 'Pandoravirus salinus', 'Tupaiid herpesvirus 1']

['Enterobacteria phage phiX174 sensu lato', 'Human papillomavirus type 16', 'Human endogenous retrovirus K113', 'Gentian ovary ring-spot virus', 'Saccharomyces cerevisiae killer virus M1']

(228, 214)

TCGA-VS-A8EK-01A

['Enterobacteria phage phiX174 sensu lato', 'Alphapapillomavirus 9', 'Human endogenous retrovirus K', 'Pandoravirus salinus', 'Shamonda virus']

['Enterobacteria phage phiX174 sensu lato', 'Human endogenous retrovirus K113', 'Encephalomyocarditis virus', 'Primula malacoides virus China/Mar2007', 'Bovine viral diarrhea virus 1']

(301, 149)

TCGA-C5-A1MQ-01A

['Enterobacteria phage phiX174 sensu lato', 'Simbu virus', 'Choristoneura occidentalis granulovirus', 'Shamonda virus', 'Alphapapillomavirus 7']

['Enterobacteria phage phiX174 sensu lato', 'Alphapapillomavirus 7', 'Human endogenous retrovirus K113', 'Saccharomyces cerevisiae killer virus M1', 'Gentian ovary ring-spot virus']

(220, 240)

TCGA-VS-A953-01A

['Enterobacteria phage phiX174 sensu lato', 'Alphapapillomavirus 9', 'Human endogenous retrovirus K', 'Pandoravirus salinus', 'Elephant endotheliotropic herpesvirus 4']

['Enterobacteria phage phiX174 sensu lato', 'Human papillomavirus type 16', 'Human endogenous retrovirus K113', 'Encephalomyocarditis virus', 'Primula malacoides virus China/Mar2007']

(203, 173)

TCGA-LP-A4AV-01A

['Pandoravirus salinus', 'Enterobacteria phage phiX174 sensu lato', 'Alphapapillomavirus 6', 'Human endogenous retrovirus K', 'Choristoneura occidentalis granulovirus']

['Enterobacteria phage phiX174 sensu lato', 'Human endogenous retrovirus K113', 'White clover cryptic virus 2', 'Hepatitis C virus', 'Primula malacoides virus China/Mar2007']

(125, 28)

TCGA-C5-A1MH-01A

['Enterobacteria phage phiX174 sensu lato', 'Simbu virus', 'Pandoravirus salinus', 'Shamonda virus', 'Alphapapillomavirus 9']

['Enterobacteria phage phiX174 sensu lato', 'Human papillomavirus type 16', 'Human endogenous retrovirus K113', 'Saccharomyces cerevisiae killer virus M1', 'White clover cryptic virus 2']

(19, 126)

TCGA-MY-A5BD-01A

['Enterobacteria phage phiX174 sensu lato', 'Human endogenous retrovirus K', 'Alphapapillomavirus 9', 'Pandoravirus salinus', 'Simbu virus']

['Enterobacteria phage phiX174 sensu lato', 'Human papillomavirus type 16', 'Human endogenous retrovirus K113', 'White clover cryptic virus 2', 'Saccharomyces cerevisiae killer virus M1']

(278, 270)

TCGA-ZJ-AAXT-01A

['Enterobacteria phage phiX174 sensu lato', 'Pandoravirus salinus', 'Human endogenous retrovirus K', 'Alphapapillomavirus 7', 'Pandoravirus dulcis']

['Enterobacteria phage phiX174 sensu lato', 'Alphapapillomavirus 7', 'Human endogenous retrovirus K113', 'Saccharomyces cerevisiae killer virus M1', 'Bovine viral diarrhea virus 1']

(97, 39)

TCGA-EK-A2PM-01A

['Enterobacteria phage phiX174 sensu lato', 'Human endogenous retrovirus K', 'Shamonda virus', 'Choristoneura occidentalis granulovirus', 'Pandoravirus salinus']

['Enterobacteria phage phiX174 sensu lato', 'Human endogenous retrovirus K113', 'Primula malacoides virus China/Mar2007', 'Red clover cryptic virus 2', 'Saccharomyces cerevisiae killer virus M1']

(239, 207)

TCGA-C5-A902-01A

['Enterobacteria phage phiX174 sensu lato', 'Alphapapillomavirus 9', 'Shamonda virus', 'Human endogenous retrovirus K', 'Choristoneura occidentalis granulovirus']

['Enterobacteria phage phiX174 sensu lato', 'Human papillomavirus type 16', 'Human endogenous retrovirus K113', 'Encephalomyocarditis virus', 'Bovine viral diarrhea virus 1']

(298, 256)

TCGA-MA-AA3X-01A

['Alphapapillomavirus 9', 'Enterobacteria phage phiX174 sensu lato', 'Human endogenous retrovirus K', 'Pandoravirus salinus', 'Simbu virus']

['Enterobacteria phage phiX174 sensu lato', 'Human papillomavirus type 16', 'Human endogenous retrovirus K113', 'Encephalomyocarditis virus', 'Primula malacoides virus China/Mar2007']

(37, 89)

TCGA-EK-A2RO-01A

['Pandoravirus salinus', 'Enterobacteria phage phiX174 sensu lato', 'Human endogenous retrovirus K', 'Macacine herpesvirus 1', 'Choristoneura occidentalis granulovirus']

['Enterobacteria phage phiX174 sensu lato', 'Human papillomavirus type 16', 'Human endogenous retrovirus K113', 'Saccharomyces cerevisiae killer virus M1', 'White clover cryptic virus 2']

(273, 264)

TCGA-VS-A9UV-01A

['Enterobacteria phage phiX174 sensu lato', 'Alphapapillomavirus 9', 'Pandoravirus salinus', 'Human endogenous retrovirus K', 'Tupaiid herpesvirus 1']

['Enterobacteria phage phiX174 sensu lato', 'Human endogenous retrovirus K113', 'Saccharomyces cerevisiae killer virus M1', 'Bovine viral diarrhea virus 1', 'Primula malacoides virus China/Mar2007']

(287, 296)

TCGA-VS-A9UJ-01A

['Enterobacteria phage phiX174 sensu lato', 'Pandoravirus salinus', 'Human endogenous retrovirus K', 'Shamonda virus', 'Choristoneura occidentalis granulovirus']

['Enterobacteria phage phiX174 sensu lato', 'Human endogenous retrovirus K113', 'Woolly monkey sarcoma virus', 'Bovine viral diarrhea virus 1', 'Saccharomyces cerevisiae killer virus M1']

(10, 145)

TCGA-JW-A5VH-01A

['Enterobacteria phage phiX174 sensu lato', 'Pandoravirus salinus', 'Human endogenous retrovirus K', 'Rosellinia necatrix partitivirus 2', 'Bacillus virus SPO1']

['Enterobacteria phage phiX174 sensu lato', 'Human endogenous retrovirus K113', 'Saccharomyces cerevisiae killer virus M1', 'Primula malacoides virus China/Mar2007', 'White clover cryptic virus 2']

(5, 140)

TCGA-JX-A5QV-01A

['Enterobacteria phage phiX174 sensu lato', 'Alphapapillomavirus 9', 'Human endogenous retrovirus K', 'Pandoravirus salinus', 'Simbu virus']

['Enterobacteria phage phiX174 sensu lato', 'Human papillomavirus type 16', 'Human endogenous retrovirus K113', 'Saccharomyces cerevisiae killer virus M1', 'White clover cryptic virus 2']

(36, 92)

TCGA-EK-A2R9-01A

['Alphapapillomavirus 9', 'Enterobacteria phage phiX174 sensu lato', 'Pandoravirus salinus', 'Human endogenous retrovirus K', 'Simbu virus']

['Enterobacteria phage phiX174 sensu lato', 'Human endogenous retrovirus K113', 'Saccharomyces cerevisiae killer virus M1', 'White clover cryptic virus 2', 'Primula malacoides virus China/Mar2007']

(270, 260)

TCGA-VS-AA62-01A

['Enterobacteria phage phiX174 sensu lato', 'Alphapapillomavirus 7', 'Human endogenous retrovirus K', 'Pandoravirus salinus', 'Tupaiid herpesvirus 1']

['Enterobacteria phage phiX174 sensu lato', 'Human endogenous retrovirus K113', 'Saccharomyces cerevisiae killer virus M1', 'Alphapapillomavirus 7', 'Bovine viral diarrhea virus 1']

(60, 84)

TCGA-FU-A3TX-01A

['Enterobacteria phage phiX174 sensu lato', 'Alphapapillomavirus 7', 'Pandoravirus salinus', 'Rosellinia necatrix partitivirus 2', 'Human endogenous retrovirus K']

['Enterobacteria phage phiX174 sensu lato', 'Alphapapillomavirus 7', 'Human endogenous retrovirus K113', 'Primula malacoides virus China/Mar2007', 'Gentian ovary ring-spot virus']

(172, 154)

TCGA-C5-A7CG-01A

['Enterobacteria phage phiX174 sensu lato', 'Alphapapillomavirus 9', 'Pandoravirus salinus', 'Human endogenous retrovirus K', 'Bacillus virus SPO1']

['Enterobacteria phage phiX174 sensu lato', 'Human endogenous retrovirus K113', 'Primula malacoides virus China/Mar2007', 'Bovine viral diarrhea virus 1', 'Saccharomyces cerevisiae killer virus M1']

(230, 220)

TCGA-VS-A8Q8-01A

['Enterobacteria phage phiX174 sensu lato', 'Pandoravirus salinus', 'Human endogenous retrovirus K', 'Tupaiid herpesvirus 1', 'Shamonda virus']

['Enterobacteria phage phiX174 sensu lato', 'Human endogenous retrovirus K113', 'Human papillomavirus type 16', 'Encephalomyocarditis virus', 'Primula malacoides virus China/Mar2007']

(231, 210)

TCGA-MY-A913-01A

['Enterobacteria phage phiX174 sensu lato', 'Alphapapillomavirus 7', 'Human endogenous retrovirus K', 'Pandoravirus salinus', 'Shamonda virus']

['Enterobacteria phage phiX174 sensu lato', 'Human endogenous retrovirus K113', 'Alphapapillomavirus 7', 'Bovine viral diarrhea virus 1', 'Encephalomyocarditis virus']

(79, 59)

TCGA-EK-A3GJ-01A

['Pandoravirus salinus', 'Enterobacteria phage phiX174 sensu lato', 'Human endogenous retrovirus K', 'Alphapapillomavirus 9', 'Rosellinia necatrix partitivirus 2']

['Enterobacteria phage phiX174 sensu lato', 'Human endogenous retrovirus K113', 'Gentian ovary ring-spot virus', 'Saccharomyces cerevisiae killer virus M1', 'White clover cryptic virus 2']

(41, 102)

TCGA-FU-A3TQ-01A

['Alphapapillomavirus 9', 'Pandoravirus salinus', 'Enterobacteria phage phiX174 sensu lato', 'Human endogenous retrovirus K', 'Simbu virus']

['Enterobacteria phage phiX174 sensu lato', 'Human papillomavirus type 16', 'Human endogenous retrovirus K113', 'Saccharomyces cerevisiae killer virus M1', 'White clover cryptic virus 2']

(187, 195)

TCGA-C5-A7CO-01A

['Enterobacteria phage phiX174 sensu lato', 'Alphapapillomavirus 9', 'Human endogenous retrovirus K', 'Pandoravirus salinus', 'Shamonda virus']

['Enterobacteria phage phiX174 sensu lato', 'Human endogenous retrovirus K113', 'Encephalomyocarditis virus', 'Primula malacoides virus China/Mar2007', 'Bovine viral diarrhea virus 1']

(242, 204)

TCGA-C5-A901-01A

['Enterobacteria phage phiX174 sensu lato', 'Alphapapillomavirus 9', 'Pandoravirus salinus', 'Macacine herpesvirus 1', 'Shamonda virus']

['Enterobacteria phage phiX174 sensu lato', 'Human papillomavirus type 16', 'Human endogenous retrovirus K113', 'Encephalomyocarditis virus', 'Primula malacoides virus China/Mar2007']

(126, 1)

TCGA-DS-A0VM-01A

['Simbu virus', 'Shamonda virus', 'Enterobacteria phage phiX174 sensu lato', 'Alphapapillomavirus 9', 'Choristoneura occidentalis granulovirus']

['Enterobacteria phage phiX174 sensu lato', 'Human papillomavirus type 16', 'Human endogenous retrovirus K113', 'Saccharomyces cerevisiae killer virus M1', 'Primula malacoides virus China/Mar2007']

(297, 255)

TCGA-UC-A7PG-01A

['Enterobacteria phage phiX174 sensu lato', 'Pandoravirus salinus', 'Human endogenous retrovirus K', 'Macacine herpesvirus 1', 'Shamonda virus']

['Enterobacteria phage phiX174 sensu lato', 'Human papillomavirus type 16', 'Human endogenous retrovirus K113', 'Encephalomyocarditis virus', 'Bovine viral diarrhea virus 1']

(7, 146)

TCGA-JW-A5VK-01A

['Enterobacteria phage phiX174 sensu lato', 'Pandoravirus salinus', 'Alphapapillomavirus 5', 'Human endogenous retrovirus K', 'Choristoneura occidentalis granulovirus']

['Enterobacteria phage phiX174 sensu lato', 'Human papillomavirus type 26', 'Human endogenous retrovirus K113', 'Saccharomyces cerevisiae killer virus M1', 'Gentian ovary ring-spot virus']

(133, 8)

TCGA-C5-A1M6-01A

['Enterobacteria phage phiX174 sensu lato', 'Simbu virus', 'Shamonda virus', 'Alphapapillomavirus 7', 'Human endogenous retrovirus K']

['Enterobacteria phage phiX174 sensu lato', 'Alphapapillomavirus 7', 'Human endogenous retrovirus K113', 'Primula malacoides virus China/Mar2007', 'Saccharomyces cerevisiae killer virus M1']

(43, 104)

TCGA-EK-A2GZ-01A

['Enterobacteria phage phiX174 sensu lato', 'Alphapapillomavirus 9', 'Human endogenous retrovirus K', 'Pandoravirus salinus', 'Simbu virus']

['Enterobacteria phage phiX174 sensu lato', 'Human endogenous retrovirus K113', 'Saccharomyces cerevisiae killer virus M1', 'Primula malacoides virus China/Mar2007', 'Red clover cryptic virus 2']

(196, 237)

TCGA-MU-A8JM-01A

['Enterobacteria phage phiX174 sensu lato', 'Alphapapillomavirus 9', 'Pandoravirus salinus', 'Human endogenous retrovirus K', 'Macacine herpesvirus 1']

['Enterobacteria phage phiX174 sensu lato', 'Human papillomavirus type 16', 'Human endogenous retrovirus K113', 'Encephalomyocarditis virus', 'Primula malacoides virus China/Mar2007']

(84, 64)

TCGA-C5-A3HD-01B

['Alphapapillomavirus 9', 'Enterobacteria phage phiX174 sensu lato', 'Human endogenous retrovirus K', 'Pandoravirus salinus', 'Rosellinia necatrix partitivirus 2']

['Human papillomavirus type 16', 'Enterobacteria phage phiX174 sensu lato', 'Human endogenous retrovirus K113', 'Primula malacoides virus China/Mar2007', 'Saccharomyces cerevisiae killer virus M1']

(170, 167)

TCGA-Q1-A6DW-01A

['Alphapapillomavirus 9', 'Enterobacteria phage phiX174 sensu lato', 'Pandoravirus salinus', 'Human endogenous retrovirus K', 'Tupaiid herpesvirus 1']

['Human papillomavirus type 16', 'Enterobacteria phage phiX174 sensu lato', 'Human endogenous retrovirus K113', 'Primula malacoides virus China/Mar2007', 'Saccharomyces cerevisiae killer virus M1']

(192, 189)

TCGA-DS-A7WF-01A

['Enterobacteria phage phiX174 sensu lato', 'Alphapapillomavirus 9', 'Human endogenous retrovirus K', 'Shamonda virus', 'Choristoneura occidentalis granulovirus']

['Enterobacteria phage phiX174 sensu lato', 'Human papillomavirus type 16', 'Human endogenous retrovirus K113', 'Woolly monkey sarcoma virus', 'Encephalomyocarditis virus']

(51, 95)

TCGA-EK-A2RJ-01A

['Enterobacteria phage phiX174 sensu lato', 'Choristoneura occidentalis granulovirus', 'Shamonda virus', 'Human endogenous retrovirus K', 'Simbu virus']

['Enterobacteria phage phiX174 sensu lato', 'Human endogenous retrovirus K113', 'White clover cryptic virus 2', 'Saccharomyces cerevisiae killer virus M1', 'Primula malacoides virus China/Mar2007']

(136, 16)

TCGA-DS-A0VN-01A

['Simbu virus', 'Shamonda virus', 'Enterobacteria phage phiX174 sensu lato', 'Choristoneura occidentalis granulovirus', 'Pandoravirus salinus']

['Enterobacteria phage phiX174 sensu lato', 'Human papillomavirus type 16', 'Human endogenous retrovirus K113', 'Saccharomyces cerevisiae killer virus M1', 'White clover cryptic virus 2']

(53, 90)

TCGA-C5-A2M1-01A

['Alphapapillomavirus 9', 'Enterobacteria phage phiX174 sensu lato', 'Human endogenous retrovirus K', 'Pandoravirus salinus', 'Rosellinia necatrix partitivirus 2']

['Enterobacteria phage phiX174 sensu lato', 'Human papillomavirus type 16', 'Human endogenous retrovirus K113', 'Saccharomyces cerevisiae killer virus M1', 'White clover cryptic virus 2']

(72, 72)

TCGA-EA-A3HR-01A

['Enterobacteria phage phiX174 sensu lato', 'Alphapapillomavirus 11', 'Pandoravirus salinus', 'Simbu virus', 'Human endogenous retrovirus K']

['Enterobacteria phage phiX174 sensu lato', 'Human endogenous retrovirus K113', 'White clover cryptic virus 2', 'Saccharomyces cerevisiae killer virus M1', 'Gentian ovary ring-spot virus']

(56, 105)

TCGA-EK-A2IP-01A

['Enterobacteria phage phiX174 sensu lato', 'Simbu virus', 'Pandoravirus salinus', 'Shamonda virus', 'Choristoneura occidentalis granulovirus']

['Enterobacteria phage phiX174 sensu lato', 'Human papillomavirus type 16', 'Human endogenous retrovirus K113', 'Saccharomyces cerevisiae killer virus M1', 'Primula malacoides virus China/Mar2007']

(138, 7)

TCGA-DR-A0ZL-01A

['Enterobacteria phage phiX174 sensu lato', 'Simbu virus', 'Alphapapillomavirus 9', 'Shamonda virus', 'Pandoravirus salinus']

['Enterobacteria phage phiX174 sensu lato', 'Human papillomavirus type 16', 'Human endogenous retrovirus K113', 'Saccharomyces cerevisiae killer virus M1', 'White clover cryptic virus 2']

(197, 231)

TCGA-C5-A7UI-01A

['Enterobacteria phage phiX174 sensu lato', 'Cardiovirus A', 'Macacine herpesvirus 1', 'Pandoravirus salinus', 'Human endogenous retrovirus K']

['Enterobacteria phage phiX174 sensu lato', 'Human endogenous retrovirus K113', 'Encephalomyocarditis virus', 'Saccharomyces cerevisiae killer virus M1', 'Red clover cryptic virus 2']

(38, 87)

TCGA-EK-A2RA-01A

['Simbu virus', 'Shamonda virus', 'Pandoravirus salinus', 'Choristoneura occidentalis granulovirus', 'Enterobacteria phage phiX174 sensu lato']

['Enterobacteria phage phiX174 sensu lato', 'Human endogenous retrovirus K113', 'White clover cryptic virus 2', 'Saccharomyces cerevisiae killer virus M1', 'Primula malacoides virus China/Mar2007']

(146, 176)

TCGA-JX-A3PZ-01A

['Enterobacteria phage phiX174 sensu lato', 'Alphapapillomavirus 7', 'Shamonda virus', 'Simbu virus', 'Choristoneura occidentalis granulovirus']

['Enterobacteria phage phiX174 sensu lato', 'Human endogenous retrovirus K113', 'Ball python nidovirus', 'Alphapapillomavirus 7', 'Bovine viral diarrhea virus 1']

(177, 185)

TCGA-FU-A770-01A

['Alphapapillomavirus 9', 'Enterobacteria phage phiX174 sensu lato', 'Human endogenous retrovirus K', 'Pandoravirus salinus', 'Shamonda virus']

['Enterobacteria phage phiX174 sensu lato', 'Human papillomavirus type 16', 'Human endogenous retrovirus K113', 'Encephalomyocarditis virus', 'Bovine viral diarrhea virus 1']

(269, 258)

TCGA-VS-A9UY-01A

['Enterobacteria phage phiX174 sensu lato', 'Alphapapillomavirus 9', 'Pandoravirus salinus', 'Human endogenous retrovirus K', 'Choristoneura occidentalis granulovirus']

['Enterobacteria phage phiX174 sensu lato', 'Human papillomavirus type 16', 'Human endogenous retrovirus K113', 'Saccharomyces cerevisiae killer virus M1', 'Bovine viral diarrhea virus 1']

(256, 284)

TCGA-ZJ-AAXD-01A

['Enterobacteria phage phiX174 sensu lato', 'Alphapapillomavirus 9', 'Pandoravirus salinus', 'Human endogenous retrovirus K', 'Choristoneura occidentalis granulovirus']

['Enterobacteria phage phiX174 sensu lato', 'Human papillomavirus type 16', 'Human endogenous retrovirus K113', 'Bovine viral diarrhea virus 1', 'Saccharomyces cerevisiae killer virus M1']

(202, 234)

TCGA-VS-A8EG-01A

['Enterobacteria phage phiX174 sensu lato', 'Pandoravirus salinus', 'Human endogenous retrovirus K', 'Alphapapillomavirus 9', 'Tupaiid herpesvirus 1']

['Enterobacteria phage phiX174 sensu lato', 'Human papillomavirus type 16', 'Human endogenous retrovirus K113', 'Bovine viral diarrhea virus 1', 'Encephalomyocarditis virus']

(1, 138)

TCGA-Q1-A5R1-01A

['Enterobacteria phage phiX174 sensu lato', 'Alphapapillomavirus 9', 'Human endogenous retrovirus K', 'Pandoravirus salinus', 'Choristoneura occidentalis granulovirus']

['Enterobacteria phage phiX174 sensu lato', 'Human papillomavirus type 16', 'Human endogenous retrovirus K113', 'Primula malacoides virus China/Mar2007', 'Red clover cryptic virus 2']

(232, 215)

TCGA-GH-A9DA-01A

['Enterobacteria phage phiX174 sensu lato', 'Alphapapillomavirus 7', 'Pandoravirus salinus', 'Shamonda virus', 'Simbu virus']

['Enterobacteria phage phiX174 sensu lato', 'Human endogenous retrovirus K113', 'Bovine viral diarrhea virus 1', 'Encephalomyocarditis virus', 'Woolly monkey sarcoma virus']

(33, 94)

TCGA-C5-A3HE-01A

['Enterobacteria phage phiX174 sensu lato', 'Simbu virus', 'Alphapapillomavirus 7', 'Human endogenous retrovirus K', 'Choristoneura occidentalis granulovirus']

['Enterobacteria phage phiX174 sensu lato', 'Alphapapillomavirus 7', 'Human endogenous retrovirus K113', 'Saccharomyces cerevisiae killer virus M1', 'White clover cryptic virus 2']

(261, 289)

TCGA-VS-A9V1-01A

['Enterobacteria phage phiX174 sensu lato', 'Alphapapillomavirus 7', 'Human endogenous retrovirus K', 'Pandoravirus salinus', 'Choristoneura occidentalis granulovirus']

['Enterobacteria phage phiX174 sensu lato', 'Human endogenous retrovirus K113', 'Bovine viral diarrhea virus 1', 'Primula malacoides virus China/Mar2007', 'Red clover cryptic virus 2']

(260, 286)

TCGA-ZJ-AAXB-01A

['Enterobacteria phage phiX174 sensu lato', 'Human endogenous retrovirus K', 'Alphapapillomavirus 7', 'Simbu virus', 'Choristoneura occidentalis granulovirus']

['Enterobacteria phage phiX174 sensu lato', 'Alphapapillomavirus 7', 'Human endogenous retrovirus K113', 'Primula malacoides virus China/Mar2007', 'Red clover cryptic virus 2']

(35, 88)

TCGA-EA-A1QS-01A

['Alphapapillomavirus 7', 'Enterobacteria phage phiX174 sensu lato', 'Pandoravirus salinus', 'Pandoravirus dulcis', 'Rosellinia necatrix partitivirus 2']

['Enterobacteria phage phiX174 sensu lato', 'Human endogenous retrovirus K113', 'Saccharomyces cerevisiae killer virus M1', 'Primula malacoides virus China/Mar2007', 'White clover cryptic virus 2']

(167, 169)

TCGA-Q1-A73P-01A

['Alphapapillomavirus 7', 'Enterobacteria phage phiX174 sensu lato', 'Human endogenous retrovirus K', 'Pandoravirus salinus', 'Bacillus virus SPO1']

['Alphapapillomavirus 7', 'Enterobacteria phage phiX174 sensu lato', 'Human endogenous retrovirus K113', 'Pestivirus giraffe-1 H138', 'Saccharomyces cerevisiae killer virus M1']

(303, 303)

TCGA-ZJ-AAXI-01A

['Enterobacteria phage phiX174 sensu lato', 'Simbu virus', 'Choristoneura occidentalis granulovirus', 'Alphapapillomavirus 9', 'Shamonda virus']

['Enterobacteria phage phiX174 sensu lato', 'Human papillomavirus type 16', 'Human endogenous retrovirus K113', 'Encephalomyocarditis virus', 'Saccharomyces cerevisiae killer virus M1']

(55, 108)

TCGA-EK-A2RK-01A

['Enterobacteria phage phiX174 sensu lato', 'Pandoravirus salinus', 'Human endogenous retrovirus K', 'Elephant endotheliotropic herpesvirus 4', 'Tupaiid herpesvirus 1']

['Enterobacteria phage phiX174 sensu lato', 'Human papillomavirus type 16', 'Human endogenous retrovirus K113', 'Saccharomyces cerevisiae killer virus M1', 'Gentian ovary ring-spot virus']

(179, 183)

TCGA-C5-A7UE-01A

['Enterobacteria phage phiX174 sensu lato', 'Pandoravirus salinus', 'Human endogenous retrovirus K', 'Alphapapillomavirus 9', 'Tupaiid herpesvirus 1']

['Enterobacteria phage phiX174 sensu lato', 'Human papillomavirus type 16', 'Human endogenous retrovirus K113', 'Encephalomyocarditis virus', 'Primula malacoides virus China/Mar2007']

(122, 22)

TCGA-C5-A1MJ-01A

['Enterobacteria phage phiX174 sensu lato', 'Alphapapillomavirus 7', 'Simbu virus', 'Human endogenous retrovirus K', 'Choristoneura occidentalis granulovirus']

['Enterobacteria phage phiX174 sensu lato', 'Alphapapillomavirus 7', 'Human endogenous retrovirus K113', 'Saccharomyces cerevisiae killer virus M1', 'White clover cryptic virus 2']

(207, 247)

TCGA-VS-A94X-01A

['Enterobacteria phage phiX174 sensu lato', 'Alphapapillomavirus 7', 'Human endogenous retrovirus K', 'Pandoravirus salinus', 'Rosellinia necatrix partitivirus 2']

['Enterobacteria phage phiX174 sensu lato', 'Human endogenous retrovirus K113', 'Primula malacoides virus China/Mar2007', 'Saccharomyces cerevisiae killer virus M1', 'Bovine viral diarrhea virus 1']

(145, 177)

TCGA-LP-A4AU-01A

['Enterobacteria phage phiX174 sensu lato', 'Simbu virus', 'Shamonda virus', 'Alphapapillomavirus 7', 'Human endogenous retrovirus K']

['Enterobacteria phage phiX174 sensu lato', 'Alphapapillomavirus 7', 'Human endogenous retrovirus K113', 'Bovine viral diarrhea virus 1', 'Saccharomyces cerevisiae killer virus M1']

(14, 128)

TCGA-EA-A5ZE-01A

['Enterobacteria phage phiX174 sensu lato', 'Human endogenous retrovirus K', 'Pandoravirus salinus', 'Tupaiid herpesvirus 1', 'Pandoravirus dulcis']

['Enterobacteria phage phiX174 sensu lato', 'Human endogenous retrovirus K113', 'Primula malacoides virus China/Mar2007', 'Gentian ovary ring-spot virus', 'Saccharomyces cerevisiae killer virus M1']

(46, 103)

TCGA-DG-A2KL-01A

['Enterobacteria phage phiX174 sensu lato', 'Alphapapillomavirus 9', 'Pandoravirus salinus', 'Human endogenous retrovirus K', 'Simbu virus']

['Enterobacteria phage phiX174 sensu lato', 'Human papillomavirus type 16', 'Human endogenous retrovirus K113', 'Saccharomyces cerevisiae killer virus M1', 'Primula malacoides virus China/Mar2007']

(280, 269)

TCGA-VS-A950-01A

['Alphapapillomavirus 9', 'Enterobacteria phage phiX174 sensu lato', 'Human endogenous retrovirus K', 'Pandoravirus salinus', 'Choristoneura occidentalis granulovirus']

['Enterobacteria phage phiX174 sensu lato', 'Human endogenous retrovirus K113', 'Saccharomyces cerevisiae killer virus M1', 'Bovine viral diarrhea virus 1', 'Human papillomavirus type 34']

(241, 213)

TCGA-C5-A8YQ-01A

['Enterobacteria phage phiX174 sensu lato', 'Simbu virus', 'Shamonda virus', 'Choristoneura occidentalis granulovirus', 'Human endogenous retrovirus K']

['Enterobacteria phage phiX174 sensu lato', 'Human endogenous retrovirus K113', 'Encephalomyocarditis virus', 'Bovine viral diarrhea virus 1', 'Saccharomyces cerevisiae killer virus M1']

(275, 278)

TCGA-VS-A957-01A

['Enterobacteria phage phiX174 sensu lato', 'Alphapapillomavirus 9', 'Human endogenous retrovirus K', 'Pandoravirus salinus', 'Choristoneura occidentalis granulovirus']

['Enterobacteria phage phiX174 sensu lato', 'Human endogenous retrovirus K113', 'Saccharomyces cerevisiae killer virus M1', 'Bovine viral diarrhea virus 1', 'Primula malacoides virus China/Mar2007']

(91, 67)

TCGA-IR-A3LK-01A

['Alphapapillomavirus 9', 'Enterobacteria phage phiX174 sensu lato', 'Pandoravirus salinus', 'Human endogenous retrovirus K', 'Macacine herpesvirus 1']

['Enterobacteria phage phiX174 sensu lato', 'Human endogenous retrovirus K113', 'Saccharomyces cerevisiae killer virus M1', 'White clover cryptic virus 2', 'Primula malacoides virus China/Mar2007']

(174, 181)

TCGA-RA-A741-01A

['Enterobacteria phage phiX174 sensu lato', 'Alphapapillomavirus 9', 'Human endogenous retrovirus K', 'Simbu virus', 'Shamonda virus']

['Enterobacteria phage phiX174 sensu lato', 'Human papillomavirus type 16', 'Human endogenous retrovirus K113', 'Encephalomyocarditis virus', 'Bovine viral diarrhea virus 1']

(218, 241)

TCGA-MA-AA3Y-01A

['Enterobacteria phage phiX174 sensu lato', 'Alphapapillomavirus 7', 'Pandoravirus salinus', 'Macacine herpesvirus 1', 'Cardiovirus A']

['Enterobacteria phage phiX174 sensu lato', 'Human endogenous retrovirus K113', 'Encephalomyocarditis virus', 'Bovine viral diarrhea virus 1', 'Primula malacoides virus China/Mar2007']

(243, 205)

TCGA-C5-A8XH-01A

['Enterobacteria phage phiX174 sensu lato', 'Human endogenous retrovirus K', 'Alphapapillomavirus 9', 'Pandoravirus salinus', 'Shamonda virus']

['Enterobacteria phage phiX174 sensu lato', 'Human papillomavirus type 16', 'Human endogenous retrovirus K113', 'Primula malacoides virus China/Mar2007', 'Red clover cryptic virus 2']

(247, 200)

TCGA-VS-A8EJ-01A

['Enterobacteria phage phiX174 sensu lato', 'Human endogenous retrovirus K', 'Choristoneura occidentalis granulovirus', 'Shamonda virus', 'Simbu virus']

['Enterobacteria phage phiX174 sensu lato', 'Human endogenous retrovirus K113', 'Primula malacoides virus China/Mar2007', 'Red clover cryptic virus 2', 'Encephalomyocarditis virus']

(264, 298)

TCGA-VS-A9UU-01A

['Enterobacteria phage phiX174 sensu lato', 'Pandoravirus salinus', 'Human endogenous retrovirus K', 'Alphapapillomavirus 9', 'Choristoneura occidentalis granulovirus']

['Enterobacteria phage phiX174 sensu lato', 'Human papillomavirus type 16', 'Human endogenous retrovirus K113', 'Saccharomyces cerevisiae killer virus M1', 'Primula malacoides virus China/Mar2007']

(163, 155)

TCGA-C5-A7CL-01A

['Enterobacteria phage phiX174 sensu lato', 'Alphapapillomavirus 9', 'Pandoravirus salinus', 'Human endogenous retrovirus K', 'Bacillus virus SPO1']

['Enterobacteria phage phiX174 sensu lato', 'Human papillomavirus type 16', 'Human endogenous retrovirus K113', 'Encephalomyocarditis virus', 'Saccharomyces cerevisiae killer virus M1']

(149, 160)

TCGA-EX-A449-01A

['Enterobacteria phage phiX174 sensu lato', 'Simbu virus', 'Alphapapillomavirus 9', 'Shamonda virus', 'Choristoneura occidentalis granulovirus']

['Enterobacteria phage phiX174 sensu lato', 'Human papillomavirus type 16', 'Human endogenous retrovirus K113', 'Bovine viral diarrhea virus 1', 'Primula malacoides virus China/Mar2007']

(39, 93)

TCGA-C5-A2LX-01A

['Alphapapillomavirus 9', 'Enterobacteria phage phiX174 sensu lato', 'Pandoravirus salinus', 'Human endogenous retrovirus K', 'Bacillus virus SPO1']

['Enterobacteria phage phiX174 sensu lato', 'Human papillomavirus type 16', 'Human endogenous retrovirus K113', 'Saccharomyces cerevisiae killer virus M1', 'White clover cryptic virus 2']

(215, 246)

TCGA-MA-AA42-01A

['Enterobacteria phage phiX174 sensu lato', 'Alphapapillomavirus 9', 'Pandoravirus salinus', 'Human endogenous retrovirus K', 'Shamonda virus']

['Enterobacteria phage phiX174 sensu lato', 'Human papillomavirus type 16', 'Human endogenous retrovirus K113', 'Encephalomyocarditis virus', 'Primula malacoides virus China/Mar2007']

(21, 127)

TCGA-MY-A5BF-01A

['Enterobacteria phage phiX174 sensu lato', 'Alphapapillomavirus 9', 'Pandoravirus salinus', 'Human endogenous retrovirus K', 'Simbu virus']

['Enterobacteria phage phiX174 sensu lato', 'Human papillomavirus type 16', 'Human endogenous retrovirus K113', 'White clover cryptic virus 2', 'Hepatitis C virus']

(69, 69)

TCGA-EK-A3GN-01A

['Enterobacteria phage phiX174 sensu lato', 'Alphapapillomavirus 9', 'Simbu virus', 'Human endogenous retrovirus K', 'Shamonda virus']

['Enterobacteria phage phiX174 sensu lato', 'Human papillomavirus type 16', 'Human endogenous retrovirus K113', 'Primula malacoides virus China/Mar2007', 'Red clover cryptic virus 2']

(85, 55)

TCGA-HG-A2PA-01A

['Enterobacteria phage phiX174 sensu lato', 'Pandoravirus salinus', 'Human endogenous retrovirus K', 'Gentian ovary ring-spot virus', 'Alphapapillomavirus 9']

['Enterobacteria phage phiX174 sensu lato', 'Human papillomavirus type 16', 'Human endogenous retrovirus K113', 'Gentian ovary ring-spot virus', 'Saccharomyces cerevisiae killer virus M1']

(59, 86)

TCGA-C5-A2LS-01A

['Enterobacteria phage phiX174 sensu lato', 'Alphapapillomavirus 9', 'Dulcamara mottle virus', 'Gentian ovary ring-spot virus', 'Simbu virus']

['Enterobacteria phage phiX174 sensu lato', 'Human papillomavirus type 16', 'Saccharomyces cerevisiae killer virus M1', 'Red clover cryptic virus 2', 'Primula malacoides virus China/Mar2007']

(156, 148)

TCGA-LP-A4AW-01A

['Enterobacteria phage phiX174 sensu lato', 'Pandoravirus salinus', 'Human endogenous retrovirus K', 'Shamonda virus', 'Rosellinia necatrix partitivirus 2']

['Enterobacteria phage phiX174 sensu lato', 'Human papillomavirus type 16', 'Human endogenous retrovirus K113', 'Saccharomyces cerevisiae killer virus M1', 'Red clover cryptic virus 2']

(205, 233)

TCGA-XS-A8TJ-01A

['Enterobacteria phage phiX174 sensu lato', 'Human endogenous retrovirus K', 'Pandoravirus salinus', 'Alphapapillomavirus 9', 'Tupaiid herpesvirus 1']

['Enterobacteria phage phiX174 sensu lato', 'Human papillomavirus type 16', 'Human endogenous retrovirus K113', 'Encephalomyocarditis virus', 'Primula malacoides virus China/Mar2007']

(103, 46)

TCGA-EK-A2RM-01A

['Enterobacteria phage phiX174 sensu lato', 'Alphapapillomavirus 7', 'Human endogenous retrovirus K', 'Simbu virus', 'Shamonda virus']

['Enterobacteria phage phiX174 sensu lato', 'Alphapapillomavirus 7', 'Human endogenous retrovirus K113', 'Red clover cryptic virus 2', 'Primula malacoides virus China/Mar2007']

(153, 159)

TCGA-DG-A2KJ-01A

['Enterobacteria phage phiX174 sensu lato', 'Simbu virus', 'Shamonda virus', 'Choristoneura occidentalis granulovirus', 'Human endogenous retrovirus K']

['Enterobacteria phage phiX174 sensu lato', 'Human endogenous retrovirus K113', 'White clover cryptic virus 2', 'Alphapapillomavirus 7', 'Saccharomyces cerevisiae killer virus M1']

(18, 134)

TCGA-DS-A5RQ-01A

['Enterobacteria phage phiX174 sensu lato', 'Pandoravirus salinus', 'Alphapapillomavirus 9', 'Human endogenous retrovirus K', 'Tupaiid herpesvirus 1']

['Enterobacteria phage phiX174 sensu lato', 'Human papillomavirus type 16', 'Human endogenous retrovirus K113', 'Saccharomyces cerevisiae killer virus M1', 'Primula malacoides virus China/Mar2007']

(117, 26)

TCGA-C5-A1ML-01A

['Enterobacteria phage phiX174 sensu lato', 'Alphapapillomavirus 9', 'Simbu virus', 'Shamonda virus', 'Human endogenous retrovirus K']

['Enterobacteria phage phiX174 sensu lato', 'Human papillomavirus type 16', 'Human endogenous retrovirus K113', 'Saccharomyces cerevisiae killer virus M1', 'White clover cryptic virus 2']

(225, 228)

TCGA-VS-A8QA-01A

['Enterobacteria phage phiX174 sensu lato', 'Alphapapillomavirus 9', 'Human endogenous retrovirus K', 'Macacine herpesvirus 1', 'Pandoravirus salinus']

['Enterobacteria phage phiX174 sensu lato', 'Human papillomavirus type 16', 'Human endogenous retrovirus K113', 'Encephalomyocarditis virus', 'Bovine viral diarrhea virus 1']

(262, 291)

TCGA-VS-A9UQ-01A

['Enterobacteria phage phiX174 sensu lato', 'Alphapapillomavirus 9', 'Human endogenous retrovirus K', 'Pandoravirus salinus', 'Pandoravirus dulcis']

['Enterobacteria phage phiX174 sensu lato', 'Human papillomavirus type 16', 'Human endogenous retrovirus K113', 'Bovine viral diarrhea virus 1', 'Saccharomyces cerevisiae killer virus M1']

(93, 42)

TCGA-EK-A2RC-01A

['Enterobacteria phage phiX174 sensu lato', 'Pandoravirus salinus', 'Human endogenous retrovirus K', 'Shamonda virus', 'Rosellinia necatrix partitivirus 2']

['Enterobacteria phage phiX174 sensu lato', 'Human papillomavirus type 16', 'Human endogenous retrovirus K113', 'Red clover cryptic virus 2', 'Saccharomyces cerevisiae killer virus M1']

(92, 57)

TCGA-EA-A3HS-01A

['Enterobacteria phage phiX174 sensu lato', 'Alphapapillomavirus 9', 'Human endogenous retrovirus K', 'Pandoravirus salinus', 'Glypta fumiferanae ichnovirus']

['Enterobacteria phage phiX174 sensu lato', 'Human papillomavirus type 16', 'Human endogenous retrovirus K113', 'Primula malacoides virus China/Mar2007', 'Saccharomyces cerevisiae killer virus M1']

(189, 192)

TCGA-UC-A7PD-01A

['Enterobacteria phage phiX174 sensu lato', 'Pandoravirus salinus', 'Shamonda virus', 'Choristoneura occidentalis granulovirus', 'Alphapapillomavirus 9']

['Enterobacteria phage phiX174 sensu lato', 'Human papillomavirus type 16', 'Human endogenous retrovirus K113', 'Encephalomyocarditis virus', 'Primula malacoides virus China/Mar2007']

(277, 273)

TCGA-VS-A9UC-01A

['Enterobacteria phage phiX174 sensu lato', 'Alphapapillomavirus 9', 'Human endogenous retrovirus K', 'Pandoravirus salinus', 'Tupaiid herpesvirus 1']

['Enterobacteria phage phiX174 sensu lato', 'Human endogenous retrovirus K113', 'Woolly monkey sarcoma virus', 'Bovine viral diarrhea virus 1', 'Primula malacoides virus China/Mar2007']

(276, 271)

TCGA-ZJ-AAXU-01A

['Enterobacteria phage phiX174 sensu lato', 'Human endogenous retrovirus K', 'Pandoravirus salinus', 'Choristoneura occidentalis granulovirus', 'Shamonda virus']

['Enterobacteria phage phiX174 sensu lato', 'Human papillomavirus type 16', 'Human endogenous retrovirus K113', 'Saccharomyces cerevisiae killer virus M1', 'Primula malacoides virus China/Mar2007']

(140, 49)

TCGA-C5-A2LY-01A

['Enterobacteria phage phiX174 sensu lato', 'Alphapapillomavirus 9', 'Pandoravirus salinus', 'Human endogenous retrovirus K', 'Choristoneura occidentalis granulovirus']

['Enterobacteria phage phiX174 sensu lato', 'Human papillomavirus type 16', 'Human endogenous retrovirus K113', 'Saccharomyces cerevisiae killer virus M1', 'Red clover cryptic virus 2']

(250, 227)

TCGA-VS-A8QM-01A

['Enterobacteria phage phiX174 sensu lato', 'Macacine herpesvirus 1', 'Cardiovirus A', 'Alphapapillomavirus 9', 'Human endogenous retrovirus K']

['Enterobacteria phage phiX174 sensu lato', 'Human papillomavirus type 16', 'Encephalomyocarditis virus', 'Human endogenous retrovirus K113', 'Bovine viral diarrhea virus 1']

(295, 263)

TCGA-VS-A9U7-01A

['Enterobacteria phage phiX174 sensu lato', 'Alphapapillomavirus 9', 'Human endogenous retrovirus K', 'Pandoravirus salinus', 'Tupaiid herpesvirus 1']

['Enterobacteria phage phiX174 sensu lato', 'Human papillomavirus type 16', 'Human endogenous retrovirus K113', 'Saccharomyces cerevisiae killer virus M1', 'Bovine viral diarrhea virus 1']

(29, 114)

TCGA-FU-A40J-01A

['Enterobacteria phage phiX174 sensu lato', 'Alphapapillomavirus 9', 'Human endogenous retrovirus K', 'Bacillus virus SPO1', 'Pandoravirus salinus']

['Enterobacteria phage phiX174 sensu lato', 'Human papillomavirus type 16', 'Human endogenous retrovirus K113', 'Saccharomyces cerevisiae killer virus M1', 'White clover cryptic virus 2']

(253, 272)

TCGA-ZJ-AAXA-01A

['Enterobacteria phage phiX174 sensu lato', 'Alphapapillomavirus 9', 'Pandoravirus salinus', 'Human endogenous retrovirus K', 'Tupaiid herpesvirus 1']

['Enterobacteria phage phiX174 sensu lato', 'Human papillomavirus type 16', 'Human endogenous retrovirus K113', 'Saccharomyces cerevisiae killer virus M1', 'Bovine viral diarrhea virus 1']

(110, 20)

TCGA-C5-A1M9-01A

['Enterobacteria phage phiX174 sensu lato', 'Alphapapillomavirus 9', 'Simbu virus', 'Shamonda virus', 'Choristoneura occidentalis granulovirus']

['Enterobacteria phage phiX174 sensu lato', 'Human papillomavirus type 16', 'Human endogenous retrovirus K113', 'Primula malacoides virus China/Mar2007', 'Red clover cryptic virus 2']

(185, 193)

TCGA-DS-A7WI-01A

['Enterobacteria phage phiX174 sensu lato', 'Macacine herpesvirus 1', 'Cardiovirus A', 'Alphapapillomavirus 9', 'Pandoravirus salinus']

['Enterobacteria phage phiX174 sensu lato', 'Human papillomavirus type 16', 'Encephalomyocarditis virus', 'Human endogenous retrovirus K113', 'Primula malacoides virus China/Mar2007']

(193, 188)

TCGA-C5-A7UH-01A

['Enterobacteria phage phiX174 sensu lato', 'Alphapapillomavirus 9', 'Shamonda virus', 'Choristoneura occidentalis granulovirus', 'Human endogenous retrovirus K']

['Enterobacteria phage phiX174 sensu lato', 'Human papillomavirus type 16', 'Human endogenous retrovirus K113', 'Encephalomyocarditis virus', 'Ball python nidovirus']

(162, 157)

TCGA-C5-A7CJ-01A

['Enterobacteria phage phiX174 sensu lato', 'Alphapapillomavirus 9', 'Pandoravirus salinus', 'Human endogenous retrovirus K', 'Bacillus virus SPO1']

['Enterobacteria phage phiX174 sensu lato', 'Human papillomavirus type 16', 'Human endogenous retrovirus K113', 'Encephalomyocarditis virus', 'Primula malacoides virus China/Mar2007']

(64, 74)

TCGA-DS-A3LQ-01A

['Enterobacteria phage phiX174 sensu lato', 'Alphapapillomavirus 5', 'Pandoravirus salinus', 'Human endogenous retrovirus K', 'Shamonda virus']

['Enterobacteria phage phiX174 sensu lato', 'Human endogenous retrovirus K113', 'Saccharomyces cerevisiae killer virus M1', 'Primula malacoides virus China/Mar2007', 'Red clover cryptic virus 2']

(3, 137)

TCGA-LP-A5U3-01A

['Enterobacteria phage phiX174 sensu lato', 'Alphapapillomavirus 9', 'Pandoravirus salinus', 'Human endogenous retrovirus K', 'Tupaiid herpesvirus 1']

['Enterobacteria phage phiX174 sensu lato', 'Human papillomavirus type 16', 'Human endogenous retrovirus K113', 'Primula malacoides virus China/Mar2007', 'Encephalomyocarditis virus']

(76, 80)

TCGA-EA-A3HQ-01A

['Enterobacteria phage phiX174 sensu lato', 'Alphapapillomavirus 9', 'Pandoravirus salinus', 'Human endogenous retrovirus K', 'Bacillus virus SPO1']

['Enterobacteria phage phiX174 sensu lato', 'Human papillomavirus type 16', 'Human endogenous retrovirus K113', 'Primula malacoides virus China/Mar2007', 'Saccharomyces cerevisiae killer virus M1']

(89, 62)

TCGA-FU-A3EO-01A

['Enterobacteria phage phiX174 sensu lato', 'Gentian ovary ring-spot virus', 'Choristoneura occidentalis granulovirus', 'Simbu virus', 'Shamonda virus']

['Enterobacteria phage phiX174 sensu lato', 'White clover cryptic virus 2', 'Saccharomyces cerevisiae killer virus M1', 'Gentian ovary ring-spot virus', 'Human papillomavirus type 16']

(118, 25)

TCGA-DS-A1OB-01A

['Enterobacteria phage phiX174 sensu lato', 'Alphapapillomavirus 9', 'Simbu virus', 'Pandoravirus salinus', 'Shamonda virus']

['Enterobacteria phage phiX174 sensu lato', 'Human endogenous retrovirus K113', 'Saccharomyces cerevisiae killer virus M1', 'Primula malacoides virus China/Mar2007', 'White clover cryptic virus 2']

(188, 194)

TCGA-WL-A834-01A

['Enterobacteria phage phiX174 sensu lato', 'Alphapapillomavirus 9', 'Shamonda virus', 'Simbu virus', 'Human endogenous retrovirus K']

['Enterobacteria phage phiX174 sensu lato', 'Human papillomavirus type 16', 'Human endogenous retrovirus K113', 'Encephalomyocarditis virus', 'Y73 sarcoma virus']

(95, 50)

TCGA-EK-A2PL-01A

['Enterobacteria phage phiX174 sensu lato', 'Choristoneura occidentalis granulovirus', 'Pandoravirus salinus', 'Shamonda virus', 'Human endogenous retrovirus K']

['Enterobacteria phage phiX174 sensu lato', 'Human endogenous retrovirus K113', 'Saccharomyces cerevisiae killer virus M1', 'Red clover cryptic virus 2', 'Primula malacoides virus China/Mar2007']

(216, 245)

TCGA-4J-AA1J-01A

['Alphapapillomavirus 7', 'Enterobacteria phage phiX174 sensu lato', 'Pandoravirus salinus', 'Human endogenous retrovirus K', 'Rosellinia necatrix partitivirus 2']

['Enterobacteria phage phiX174 sensu lato', 'Alphapapillomavirus 7', 'Human endogenous retrovirus K113', 'Encephalomyocarditis virus', 'Bovine viral diarrhea virus 1']

(252, 275)

TCGA-VS-A9UP-01A

['Enterobacteria phage phiX174 sensu lato', 'Alphapapillomavirus 7', 'Human endogenous retrovirus K', 'Pandoravirus salinus', 'Shamonda virus']

['Enterobacteria phage phiX174 sensu lato', 'Alphapapillomavirus 7', 'Human endogenous retrovirus K113', 'Saccharomyces cerevisiae killer virus M1', 'Bovine viral diarrhea virus 1']

(244, 206)

TCGA-C5-A8ZZ-01A

['Enterobacteria phage phiX174 sensu lato', 'Human endogenous retrovirus K', 'Simbu virus', 'Shamonda virus', 'Choristoneura occidentalis granulovirus']

['Enterobacteria phage phiX174 sensu lato', 'Human papillomavirus type 16', 'Human endogenous retrovirus K113', 'Encephalomyocarditis virus', 'Bovine viral diarrhea virus 1']

(221, 226)

TCGA-ZJ-A8QR-01A

['Enterobacteria phage phiX174 sensu lato', 'Alphapapillomavirus 9', 'Human endogenous retrovirus K', 'Pandoravirus salinus', 'Macacine herpesvirus 1']

['Enterobacteria phage phiX174 sensu lato', 'Human papillomavirus type 16', 'Human endogenous retrovirus K113', 'Encephalomyocarditis virus', 'Saccharomyces cerevisiae killer virus M1']

(200, 235)

TCGA-C5-A7X5-01A

['Enterobacteria phage phiX174 sensu lato', 'Alphapapillomavirus 9', 'Human endogenous retrovirus K', 'Pandoravirus salinus', 'Tupaiid herpesvirus 1']

['Enterobacteria phage phiX174 sensu lato', 'Human endogenous retrovirus K113', 'Bovine viral diarrhea virus 1', 'Primula malacoides virus China/Mar2007', 'Encephalomyocarditis virus']

(147, 163)

TCGA-IR-A3LI-01A

['Enterobacteria phage phiX174 sensu lato', 'Choristoneura occidentalis granulovirus', 'Human endogenous retrovirus K', 'Shamonda virus', 'Alphapapillomavirus 9']

['Enterobacteria phage phiX174 sensu lato', 'Human papillomavirus type 16', 'Human endogenous retrovirus K113', 'Encephalomyocarditis virus', 'White clover cryptic virus 2']

(258, 280)

TCGA-VS-A9V2-01A

['Enterobacteria phage phiX174 sensu lato', 'Human endogenous retrovirus K', 'Alphapapillomavirus 9', 'Pandoravirus salinus', 'Choristoneura occidentalis granulovirus']

['Enterobacteria phage phiX174 sensu lato', 'Human papillomavirus type 16', 'Human endogenous retrovirus K113', 'Primula malacoides virus China/Mar2007', 'Bovine viral diarrhea virus 1']

(68, 70)

TCGA-FU-A3NI-01A

['Enterobacteria phage phiX174 sensu lato', 'Alphapapillomavirus 9', 'Human endogenous retrovirus K', 'Pandoravirus salinus', 'Glypta fumiferanae ichnovirus']

['Enterobacteria phage phiX174 sensu lato', 'Human papillomavirus type 16', 'Human endogenous retrovirus K113', 'Saccharomyces cerevisiae killer virus M1', 'Primula malacoides virus China/Mar2007']

(212, 254)

TCGA-VS-A952-01A

['Enterobacteria phage phiX174 sensu lato', 'Alphapapillomavirus 9', 'Human endogenous retrovirus K', 'Shamonda virus', 'Choristoneura occidentalis granulovirus']

['Enterobacteria phage phiX174 sensu lato', 'Human papillomavirus type 16', 'Human endogenous retrovirus K113', 'Primula malacoides virus China/Mar2007', 'Bovine viral diarrhea virus 1']

(0, 135)

TCGA-Q1-A5R3-01A

['Enterobacteria phage phiX174 sensu lato', 'Pandoravirus salinus', 'Human endogenous retrovirus K', 'Simbu virus', 'Tupaiid herpesvirus 1']

['Enterobacteria phage phiX174 sensu lato', 'Human endogenous retrovirus K113', 'Alphapapillomavirus 7', 'Gentian ovary ring-spot virus', 'Saccharomyces cerevisiae killer virus M1']

(208, 248)

TCGA-C5-A7XC-01A

['Enterobacteria phage phiX174 sensu lato', 'Pandoravirus salinus', 'Human endogenous retrovirus K', 'Alphapapillomavirus 9', 'Bacillus virus SPO1']

['Enterobacteria phage phiX174 sensu lato', 'Human papillomavirus type 16', 'Human endogenous retrovirus K113', 'Primula malacoides virus China/Mar2007', 'Red clover cryptic virus 2']

(144, 9)

TCGA-DS-A0VL-01A

['Simbu virus', 'Enterobacteria phage phiX174 sensu lato', 'Alphapapillomavirus 9', 'Shamonda virus', 'Choristoneura occidentalis granulovirus']

['Enterobacteria phage phiX174 sensu lato', 'Human papillomavirus type 16', 'Human endogenous retrovirus K113', 'Saccharomyces cerevisiae killer virus M1', 'Primula malacoides virus China/Mar2007']

(61, 85)

TCGA-EA-A3QD-01A

['Enterobacteria phage phiX174 sensu lato', 'Alphapapillomavirus 9', 'Gentian ovary ring-spot virus', 'Pandoravirus salinus', 'Human endogenous retrovirus K']

['Enterobacteria phage phiX174 sensu lato', 'Human papillomavirus type 16', 'Gentian ovary ring-spot virus', 'Saccharomyces cerevisiae killer virus M1', 'Human endogenous retrovirus K113']

(151, 164)

TCGA-EA-A43B-01A

['Enterobacteria phage phiX174 sensu lato', 'Alphapapillomavirus 9', 'Pandoravirus salinus', 'Human endogenous retrovirus K', 'Elephant endotheliotropic herpesvirus 4']

['Enterobacteria phage phiX174 sensu lato', 'Human papillomavirus type 16', 'Human endogenous retrovirus K113', 'White clover cryptic virus 2', 'Saccharomyces cerevisiae killer virus M1']

(32, 115)

TCGA-EA-A3Y4-01A

['Enterobacteria phage phiX174 sensu lato', 'Pandoravirus salinus', 'Elephant endotheliotropic herpesvirus 4', 'Human endogenous retrovirus K', 'Rosellinia necatrix partitivirus 2']

['Enterobacteria phage phiX174 sensu lato', 'Human endogenous retrovirus K113', 'Saccharomyces cerevisiae killer virus M1', 'Red clover cryptic virus 2', 'Primula malacoides virus China/Mar2007']

(94, 45)

TCGA-C5-A2LT-01A

['Enterobacteria phage phiX174 sensu lato', 'Pandoravirus salinus', 'Shamonda virus', 'Alphapapillomavirus 7', 'Choristoneura occidentalis granulovirus']

['Enterobacteria phage phiX174 sensu lato', 'Human endogenous retrovirus K113', 'Saccharomyces cerevisiae killer virus M1', 'Red clover cryptic virus 2', 'Primula malacoides virus China/Mar2007']

(28, 118)

TCGA-HM-A4S6-01A

['Enterobacteria phage phiX174 sensu lato', 'Alphapapillomavirus 9', 'Pandoravirus salinus', 'Human endogenous retrovirus K', 'Shamonda virus']

['Enterobacteria phage phiX174 sensu lato', 'Human papillomavirus type 16', 'Human endogenous retrovirus K113', 'Saccharomyces cerevisiae killer virus M1', 'White clover cryptic virus 2']

(293, 267)

TCGA-ZJ-AAXN-01A

['Alphapapillomavirus 7', 'Enterobacteria phage phiX174 sensu lato', 'Human endogenous retrovirus K', 'Pandoravirus salinus', 'Choristoneura occidentalis granulovirus']

['Enterobacteria phage phiX174 sensu lato', 'Human endogenous retrovirus K113', 'Saccharomyces cerevisiae killer virus M1', 'Primula malacoides virus China/Mar2007', 'Bovine viral diarrhea virus 1']

(201, 236)

TCGA-VS-A8EH-01A

['Enterobacteria phage phiX174 sensu lato', 'Human endogenous retrovirus K', 'Pandoravirus salinus', 'Alphapapillomavirus 9', 'Shamonda virus']

['Enterobacteria phage phiX174 sensu lato', 'Human papillomavirus type 16', 'Human endogenous retrovirus K113', 'Encephalomyocarditis virus', 'Saccharomyces cerevisiae killer virus M1']

(17, 130)

TCGA-EA-A5O9-01A

['Enterobacteria phage phiX174 sensu lato', 'Human endogenous retrovirus K', 'Alphapapillomavirus 9', 'Pandoravirus salinus', 'Tupaiid herpesvirus 1']

['Enterobacteria phage phiX174 sensu lato', 'Human papillomavirus type 16', 'Human endogenous retrovirus K113', 'Saccharomyces cerevisiae killer virus M1', 'Primula malacoides virus China/Mar2007']

(83, 63)

TCGA-EK-A2RN-01A

['Choristoneura occidentalis granulovirus', 'Enterobacteria phage phiX174 sensu lato', 'Pandoravirus salinus', 'Simbu virus', 'Shamonda virus']

['Enterobacteria phage phiX174 sensu lato', 'Human papillomavirus type 16', 'Saccharomyces cerevisiae killer virus M1', 'White clover cryptic virus 2', 'Red clover cryptic virus 2']

(108, 19)

TCGA-C5-A1M7-01A

['Enterobacteria phage phiX174 sensu lato', 'Pandoravirus salinus', 'Simbu virus', 'Shamonda virus', 'Alphapapillomavirus 7']

['Enterobacteria phage phiX174 sensu lato', 'Alphapapillomavirus 7', 'Human endogenous retrovirus K113', 'Saccharomyces cerevisiae killer virus M1', 'Primula malacoides virus China/Mar2007']

(289, 299)

TCGA-VS-A9UI-01A

['Enterobacteria phage phiX174 sensu lato', 'Alphapapillomavirus 9', 'Pandoravirus salinus', 'Human endogenous retrovirus K', 'Tupaiid herpesvirus 1']

['Enterobacteria phage phiX174 sensu lato', 'Human endogenous retrovirus K113', 'Encephalomyocarditis virus', 'Saccharomyces cerevisiae killer virus M1', 'Bovine viral diarrhea virus 1']

(182, 178)

TCGA-C5-A7CH-01A

['Enterobacteria phage phiX174 sensu lato', 'Alphapapillomavirus 9', 'Pandoravirus salinus', 'Shamonda virus', 'Human endogenous retrovirus K']

['Enterobacteria phage phiX174 sensu lato', 'Human papillomavirus type 16', 'Human endogenous retrovirus K113', 'Saccharomyces cerevisiae killer virus M1', 'Bovine viral diarrhea virus 1']

(47, 110)

TCGA-FU-A23K-01A

['Enterobacteria phage phiX174 sensu lato', 'Alphapapillomavirus 7', 'Simbu virus', 'Choristoneura occidentalis granulovirus', 'Elephant endotheliotropic herpesvirus 4']

['Enterobacteria phage phiX174 sensu lato', 'Alphapapillomavirus 7', 'Saccharomyces cerevisiae killer virus M1', 'White clover cryptic virus 2', 'Human endogenous retrovirus K113']

(139, 116)

TCGA-EA-A411-01A

['Enterobacteria phage phiX174 sensu lato', 'Alphapapillomavirus 9', 'Pandoravirus salinus', 'Human endogenous retrovirus K', 'Pandoravirus dulcis']

['Enterobacteria phage phiX174 sensu lato', 'Human papillomavirus type 16', 'Human endogenous retrovirus K113', 'Saccharomyces cerevisiae killer virus M1', 'Primula malacoides virus China/Mar2007']

(234, 212)

TCGA-C5-A8XJ-01A

['Enterobacteria phage phiX174 sensu lato', 'Alphapapillomavirus 9', 'Human endogenous retrovirus K', 'Shamonda virus', 'Choristoneura occidentalis granulovirus']

['Enterobacteria phage phiX174 sensu lato', 'Human endogenous retrovirus K113', 'Y73 sarcoma virus', 'Bovine viral diarrhea virus 1', 'Saccharomyces cerevisiae killer virus M1']

(219, 242)

TCGA-MA-AA3Z-01A

['Enterobacteria phage phiX174 sensu lato', 'Alphapapillomavirus 9', 'Human endogenous retrovirus K', 'Pandoravirus salinus', 'Tupaiid herpesvirus 1']

['Enterobacteria phage phiX174 sensu lato', 'Human papillomavirus type 16', 'Human endogenous retrovirus K113', 'Primula malacoides virus China/Mar2007', 'Bovine viral diarrhea virus 1']

(63, 81)

TCGA-EA-A3QE-01A

['Enterobacteria phage phiX174 sensu lato', 'Alphapapillomavirus 9', 'Human endogenous retrovirus K', 'Pandoravirus salinus', 'Bacillus virus SPO1']

['Enterobacteria phage phiX174 sensu lato', 'Human papillomavirus type 16', 'Human endogenous retrovirus K113', 'Primula malacoides virus China/Mar2007', 'Saccharomyces cerevisiae killer virus M1']

(274, 268)

TCGA-VS-A9V0-01A

['Enterobacteria phage phiX174 sensu lato', 'Human endogenous retrovirus K', 'Pandoravirus salinus', 'Choristoneura occidentalis granulovirus', 'Simbu virus']

['Enterobacteria phage phiX174 sensu lato', 'Human endogenous retrovirus K113', 'Bovine viral diarrhea virus 1', 'Primula malacoides virus China/Mar2007', 'Human papillomavirus type 16']

(157, 172)

TCGA-R2-A69V-01A

['Enterobacteria phage phiX174 sensu lato', 'Human endogenous retrovirus K', 'Pandoravirus salinus', 'Alphapapillomavirus 7', 'Rosellinia necatrix partitivirus 2']

['Enterobacteria phage phiX174 sensu lato', 'Human endogenous retrovirus K113', 'Saccharomyces cerevisiae killer virus M1', 'Primula malacoides virus China/Mar2007', 'Red clover cryptic virus 2']

(52, 98)

TCGA-C5-A2LV-01A

['Alphapapillomavirus 9', 'Pandoravirus salinus', 'Enterobacteria phage phiX174 sensu lato', 'Simbu virus', 'Human endogenous retrovirus K']

['Enterobacteria phage phiX174 sensu lato', 'Human papillomavirus type 16', 'Human endogenous retrovirus K113', 'White clover cryptic virus 2', 'Saccharomyces cerevisiae killer virus M1']

(102, 44)

TCGA-C5-A2M2-01A

['Enterobacteria phage phiX174 sensu lato', 'Alphapapillomavirus 9', 'Human endogenous retrovirus K', 'Pandoravirus salinus', 'Simbu virus']

['Enterobacteria phage phiX174 sensu lato', 'Human papillomavirus type 16', 'Human endogenous retrovirus K113', 'Red clover cryptic virus 2', 'White clover cryptic virus 2']

(257, 279)

TCGA-VS-A9UO-01A

['Enterobacteria phage phiX174 sensu lato', 'Alphapapillomavirus 7', 'Human endogenous retrovirus K', 'Simbu virus', 'Shamonda virus']

['Enterobacteria phage phiX174 sensu lato', 'Alphapapillomavirus 7', 'Human endogenous retrovirus K113', 'Saccharomyces cerevisiae killer virus M1', 'Bovine viral diarrhea virus 1']

(227, 225)

TCGA-VS-A8Q9-01A

['Alphapapillomavirus 5', 'Enterobacteria phage phiX174 sensu lato', 'Human endogenous retrovirus K', 'Macacine herpesvirus 1', 'Shamonda virus']

['Enterobacteria phage phiX174 sensu lato', 'Human endogenous retrovirus K113', 'Encephalomyocarditis virus', 'Saccharomyces cerevisiae killer virus M1', 'Primula malacoides virus China/Mar2007']

(291, 265)

TCGA-MA-AA43-01A

['Enterobacteria phage phiX174 sensu lato', 'Pandoravirus salinus', 'Pandoravirus dulcis', 'Alphapapillomavirus 7', 'Tomato aspermy virus']

['Enterobacteria phage phiX174 sensu lato', 'Alphapapillomavirus 7', 'Human endogenous retrovirus K113', 'Woolly monkey sarcoma virus', 'Primula malacoides virus China/Mar2007']

(11, 136)

TCGA-JW-A5VG-01A

['Enterobacteria phage phiX174 sensu lato', 'Alphapapillomavirus 7', 'Pandoravirus salinus', 'Human endogenous retrovirus K', 'Simbu virus']

['Enterobacteria phage phiX174 sensu lato', 'Human endogenous retrovirus K113', 'Primula malacoides virus China/Mar2007', 'Saccharomyces cerevisiae killer virus M1', 'Gentian ovary ring-spot virus']

(130, 6)

TCGA-C5-A1BI-01B

['Alphapapillomavirus 9', 'Enterobacteria phage phiX174 sensu lato', 'Pandoravirus salinus', 'Simbu virus', 'Human endogenous retrovirus K']

['Human papillomavirus type 16', 'Enterobacteria phage phiX174 sensu lato', 'Human endogenous retrovirus K113', 'Primula malacoides virus China/Mar2007', 'White clover cryptic virus 2']

(223, 222)

TCGA-ZJ-A8QO-01A

['Enterobacteria phage phiX174 sensu lato', 'Alphapapillomavirus 9', 'Human endogenous retrovirus K', 'Shamonda virus', 'Choristoneura occidentalis granulovirus']

['Enterobacteria phage phiX174 sensu lato', 'Human endogenous retrovirus K113', 'Encephalomyocarditis virus', 'Bovine viral diarrhea virus 1', 'Saccharomyces cerevisiae killer virus M1']

(132, 13)

TCGA-C5-A1BL-01A

['Enterobacteria phage phiX174 sensu lato', 'Alphapapillomavirus 9', 'Pandoravirus salinus', 'Human endogenous retrovirus K', 'Simbu virus']

['Enterobacteria phage phiX174 sensu lato', 'Human papillomavirus type 16', 'Human endogenous retrovirus K113', 'Saccharomyces cerevisiae killer virus M1', 'Primula malacoides virus China/Mar2007']

(12, 133)

TCGA-FU-A5XV-01A

['Enterobacteria phage phiX174 sensu lato', 'Pandoravirus salinus', 'Human endogenous retrovirus K', 'Alphapapillomavirus 7', 'Choristoneura occidentalis granulovirus']

['Enterobacteria phage phiX174 sensu lato', 'Alphapapillomavirus 7', 'Human endogenous retrovirus K113', 'Bovine viral diarrhea virus 1', 'Primula malacoides virus China/Mar2007']

(181, 179)

TCGA-Q1-A73R-01A

['Alphapapillomavirus 9', 'Enterobacteria phage phiX174 sensu lato', 'Human endogenous retrovirus K', 'Pandoravirus dulcis', 'Pandoravirus salinus']

['Enterobacteria phage phiX174 sensu lato', 'Human papillomavirus type 16', 'Human endogenous retrovirus K113', 'Bovine viral diarrhea virus 1', 'Woolly monkey sarcoma virus']

(98, 47)

TCGA-EK-A2PK-01A

['Enterobacteria phage phiX174 sensu lato', 'Choristoneura occidentalis granulovirus', 'Alphapapillomavirus 7', 'Shamonda virus', 'Human endogenous retrovirus K']

['Enterobacteria phage phiX174 sensu lato', 'Alphapapillomavirus 7', 'Human endogenous retrovirus K113', 'Red clover cryptic virus 2', 'Primula malacoides virus China/Mar2007']

(168, 168)

TCGA-Q1-A6DT-01A

['Alphapapillomavirus 9', 'Enterobacteria phage phiX174 sensu lato', 'Human endogenous retrovirus K', 'Pandoravirus salinus', 'Rosellinia necatrix partitivirus 2']

['Human papillomavirus type 16', 'Enterobacteria phage phiX174 sensu lato', 'Human endogenous retrovirus K113', 'Primula malacoides virus China/Mar2007', 'Saccharomyces cerevisiae killer virus M1']

(175, 186)

TCGA-HM-A6W2-01A

['Enterobacteria phage phiX174 sensu lato', 'Human endogenous retrovirus K', 'Choristoneura occidentalis granulovirus', 'Shamonda virus', 'Elephant endotheliotropic herpesvirus 4']

['Enterobacteria phage phiX174 sensu lato', 'Human endogenous retrovirus K113', 'Bovine viral diarrhea virus 1', 'Encephalomyocarditis virus', 'Primula malacoides virus China/Mar2007']

(183, 198)

TCGA-UC-A7PF-01A

['Enterobacteria phage phiX174 sensu lato', 'Alphapapillomavirus 9', 'Macacine herpesvirus 1', 'Human endogenous retrovirus K', 'Cardiovirus A']

['Enterobacteria phage phiX174 sensu lato', 'Human papillomavirus type 16', 'Human endogenous retrovirus K113', 'Encephalomyocarditis virus', 'Primula malacoides virus China/Mar2007']

(240, 217)

TCGA-C5-A8XK-01A

['Enterobacteria phage phiX174 sensu lato', 'Simbu virus', 'Shamonda virus', 'Alphapapillomavirus 9', 'Choristoneura occidentalis granulovirus']

['Enterobacteria phage phiX174 sensu lato', 'Human papillomavirus type 16', 'Human endogenous retrovirus K113', 'Encephalomyocarditis virus', 'Bovine viral diarrhea virus 1']

(224, 229)

TCGA-ZJ-A8QQ-01A

['Enterobacteria phage phiX174 sensu lato', 'Macacine herpesvirus 1', 'Cardiovirus A', 'Alphapapillomavirus 7', 'Human endogenous retrovirus K']

['Enterobacteria phage phiX174 sensu lato', 'Encephalomyocarditis virus', 'Human endogenous retrovirus K113', 'Saccharomyces cerevisiae killer virus M1', 'Bovine viral diarrhea virus 1']

(70, 76)

TCGA-EA-A3HT-01A

['Enterobacteria phage phiX174 sensu lato', 'Alphapapillomavirus 9', 'Pandoravirus salinus', 'Human endogenous retrovirus K', 'Rosellinia necatrix partitivirus 2']

['Enterobacteria phage phiX174 sensu lato', 'Human papillomavirus type 16', 'Human endogenous retrovirus K113', 'Saccharomyces cerevisiae killer virus M1', 'Primula malacoides virus China/Mar2007']

(99, 40)

TCGA-EK-A2R8-01A

['Alphapapillomavirus 7', 'Enterobacteria phage phiX174 sensu lato', 'Shamonda virus', 'Simbu virus', 'Choristoneura occidentalis granulovirus']

['Enterobacteria phage phiX174 sensu lato', 'Human endogenous retrovirus K113', 'Saccharomyces cerevisiae killer virus M1', 'Red clover cryptic virus 2', 'Primula malacoides virus China/Mar2007']

(13, 132)

TCGA-EA-A5ZF-01A

['Enterobacteria phage phiX174 sensu lato', 'Human endogenous retrovirus K', 'Alphapapillomavirus 7', 'Pandoravirus salinus', 'Simbu virus']

['Enterobacteria phage phiX174 sensu lato', 'Alphapapillomavirus 7', 'Human endogenous retrovirus K113', 'Primula malacoides virus China/Mar2007', 'Bovine viral diarrhea virus 1']

(30, 113)

TCGA-IR-A3LB-01A

['Enterobacteria phage phiX174 sensu lato', 'Alphapapillomavirus 7', 'Pandoravirus salinus', 'Pandoravirus dulcis', 'Human endogenous retrovirus K']

['Enterobacteria phage phiX174 sensu lato', 'Human endogenous retrovirus K113', 'Saccharomyces cerevisiae killer virus M1', 'Gentian ovary ring-spot virus', 'White clover cryptic virus 2']

(222, 221)

TCGA-C5-A8XI-01A

['Enterobacteria phage phiX174 sensu lato', 'Alphapapillomavirus 9', 'Human endogenous retrovirus K', 'Macacine herpesvirus 1', 'Cardiovirus A']

['Enterobacteria phage phiX174 sensu lato', 'Human endogenous retrovirus K113', 'Encephalomyocarditis virus', 'Bovine viral diarrhea virus 1', 'Saccharomyces cerevisiae killer virus M1']

(113, 24)

TCGA-C5-A1MN-01A

['Enterobacteria phage phiX174 sensu lato', 'Simbu virus', 'Alphapapillomavirus 9', 'Shamonda virus', 'Choristoneura occidentalis granulovirus']

['Enterobacteria phage phiX174 sensu lato', 'Human papillomavirus type 16', 'Saccharomyces cerevisiae killer virus M1', 'Human endogenous retrovirus K113', 'White clover cryptic virus 2']

(180, 182)

TCGA-C5-A7CM-01A

['Enterobacteria phage phiX174 sensu lato', 'Simbu virus', 'Shamonda virus', 'Choristoneura occidentalis granulovirus', 'Human endogenous retrovirus K']

['Enterobacteria phage phiX174 sensu lato', 'Alphapapillomavirus 7', 'Human endogenous retrovirus K113', 'Encephalomyocarditis virus', 'Bovine viral diarrhea virus 1']

(246, 203)

TCGA-2W-A8YY-01A

['Enterobacteria phage phiX174 sensu lato', 'Macacine herpesvirus 1', 'Human endogenous retrovirus K', 'Cardiovirus A', 'Shamonda virus']

['Enterobacteria phage phiX174 sensu lato', 'Human endogenous retrovirus K113', 'Encephalomyocarditis virus', 'Bovine viral diarrhea virus 1', 'Primula malacoides virus China/Mar2007']

(210, 252)

TCGA-MA-AA3W-01A

['Enterobacteria phage phiX174 sensu lato', 'Alphapapillomavirus 9', 'Human endogenous retrovirus K', 'Pandoravirus salinus', 'Choristoneura occidentalis granulovirus']

['Enterobacteria phage phiX174 sensu lato', 'Human papillomavirus type 16', 'Human endogenous retrovirus K113', 'Primula malacoides virus China/Mar2007', 'Saccharomyces cerevisiae killer virus M1']

(271, 262)

TCGA-VS-A9UM-01A

['Enterobacteria phage phiX174 sensu lato', 'Pandoravirus salinus', 'Human endogenous retrovirus K', 'Alphapapillomavirus 9', 'Bacillus virus SPO1']

['Enterobacteria phage phiX174 sensu lato', 'Human papillomavirus type 16', 'Human endogenous retrovirus K113', 'Bovine viral diarrhea virus 1', 'Saccharomyces cerevisiae killer virus M1']

(90, 66)

TCGA-C5-A1BQ-01C

['Enterobacteria phage phiX174 sensu lato', 'Alphapapillomavirus 9', 'Pandoravirus salinus', 'Human endogenous retrovirus K', 'Tupaiid herpesvirus 1']

['Enterobacteria phage phiX174 sensu lato', 'Human endogenous retrovirus K113', 'Saccharomyces cerevisiae killer virus M1', 'Primula malacoides virus China/Mar2007', 'White clover cryptic virus 2']

(71, 79)

TCGA-JX-A3Q8-01A

['Enterobacteria phage phiX174 sensu lato', 'Alphapapillomavirus 9', 'Simbu virus', 'Human endogenous retrovirus K', 'Rosellinia necatrix partitivirus 2']

['Enterobacteria phage phiX174 sensu lato', 'Human papillomavirus type 16', 'Human endogenous retrovirus K113', 'Saccharomyces cerevisiae killer virus M1', 'White clover cryptic virus 2']

(265, 292)

TCGA-VS-A9V4-01A

['Enterobacteria phage phiX174 sensu lato', 'Human endogenous retrovirus K', 'Bacillus virus SPO1', 'Vicia cryptic virus', 'Hepatitis C virus']

['Enterobacteria phage phiX174 sensu lato', 'Human endogenous retrovirus K113', 'Saccharomyces cerevisiae killer virus M1', 'Primula malacoides virus China/Mar2007', 'Bovine viral diarrhea virus 1']

(299, 30)

TCGA-DS-A1OA-01A

['Enterobacteria phage phiX174 sensu lato', 'Simbu virus', 'Shamonda virus', 'Choristoneura occidentalis granulovirus', 'Alphapapillomavirus 9']

['Enterobacteria phage phiX174 sensu lato', 'Human endogenous retrovirus K113', 'Saccharomyces cerevisiae killer virus M1', 'White clover cryptic virus 2', 'Primula malacoides virus China/Mar2007']

(106, 37)

TCGA-EA-A1QT-01A

['Enterobacteria phage phiX174 sensu lato', 'Alphapapillomavirus 9', 'Simbu virus', 'Human endogenous retrovirus K', 'Shamonda virus']

['Enterobacteria phage phiX174 sensu lato', 'Human papillomavirus type 16', 'Human endogenous retrovirus K113', 'Saccharomyces cerevisiae killer virus M1', 'White clover cryptic virus 2']

(115, 33)

TCGA-C5-A1MP-01A

['Enterobacteria phage phiX174 sensu lato', 'Simbu virus', 'Alphapapillomavirus 9', 'Shamonda virus', 'Choristoneura occidentalis granulovirus']

['Enterobacteria phage phiX174 sensu lato', 'Human papillomavirus type 16', 'Human endogenous retrovirus K113', 'Saccharomyces cerevisiae killer virus M1', 'Primula malacoides virus China/Mar2007']

(74, 75)

TCGA-EK-A3GK-01A

['Enterobacteria phage phiX174 sensu lato', 'Alphapapillomavirus 9', 'Pandoravirus salinus', 'Human endogenous retrovirus K', 'Vicia cryptic virus']

['Enterobacteria phage phiX174 sensu lato', 'Human papillomavirus type 16', 'Human endogenous retrovirus K113', 'Saccharomyces cerevisiae killer virus M1', 'White clover cryptic virus 2']

(116, 32)

TCGA-DS-A1OC-01A

['Enterobacteria phage phiX174 sensu lato', 'Alphapapillomavirus 9', 'Pandoravirus salinus', 'Simbu virus', 'Human endogenous retrovirus K']

['Enterobacteria phage phiX174 sensu lato', 'Human papillomavirus type 16', 'Human endogenous retrovirus K113', 'Saccharomyces cerevisiae killer virus M1', 'White clover cryptic virus 2']

(165, 171)

TCGA-Q1-A73O-01A

['Alphapapillomavirus 7', 'Enterobacteria phage phiX174 sensu lato', 'Pandoravirus salinus', 'Human endogenous retrovirus K', 'Rosellinia necatrix partitivirus 2']

['Enterobacteria phage phiX174 sensu lato', 'Alphapapillomavirus 7', 'Human endogenous retrovirus K113', 'Saccharomyces cerevisiae killer virus M1', 'Primula malacoides virus China/Mar2007']

(120, 23)

TCGA-DS-A1OD-01A

['Enterobacteria phage phiX174 sensu lato', 'Simbu virus', 'Human endogenous retrovirus K', 'Alphapapillomavirus 9', 'Shamonda virus']

['Enterobacteria phage phiX174 sensu lato', 'Human papillomavirus type 16', 'Human endogenous retrovirus K113', 'Saccharomyces cerevisiae killer virus M1', 'White clover cryptic virus 2']

(50, 100)

TCGA-EK-A2RB-01A

['Alphapapillomavirus 9', 'Human endogenous retrovirus K', 'Enterobacteria phage phiX174 sensu lato', 'Pandoravirus salinus', 'Choristoneura occidentalis granulovirus']

['Enterobacteria phage phiX174 sensu lato', 'Human papillomavirus type 16', 'Human endogenous retrovirus K113', 'Saccharomyces cerevisiae killer virus M1', 'White clover cryptic virus 2']

(217, 243)

TCGA-VS-A954-01A

['Enterobacteria phage phiX174 sensu lato', 'Alphapapillomavirus 9', 'Pandoravirus salinus', 'Human endogenous retrovirus K', 'Shamonda virus']

['Enterobacteria phage phiX174 sensu lato', 'Human endogenous retrovirus K113', 'Encephalomyocarditis virus', 'Primula malacoides virus China/Mar2007', 'Saccharomyces cerevisiae killer virus M1']

(135, 11)

TCGA-C5-A1BJ-01A

['Enterobacteria phage phiX174 sensu lato', 'Alphapapillomavirus 9', 'Pandoravirus salinus', 'Human endogenous retrovirus K', 'Simbu virus']

['Enterobacteria phage phiX174 sensu lato', 'Human papillomavirus type 16', 'Human endogenous retrovirus K113', 'White clover cryptic virus 2', 'Saccharomyces cerevisiae killer virus M1']

(272, 261)

TCGA-VS-A9V5-01A

['Enterobacteria phage phiX174 sensu lato', 'Alphapapillomavirus 9', 'Human endogenous retrovirus K', 'Pandoravirus salinus', 'Choristoneura occidentalis granulovirus']

['Enterobacteria phage phiX174 sensu lato', 'Human papillomavirus type 16', 'Human endogenous retrovirus K113', 'Bovine viral diarrhea virus 1', 'Primula malacoides virus China/Mar2007']

(166, 166)

TCGA-Q1-A73Q-01A

['Alphapapillomavirus 9', 'Enterobacteria phage phiX174 sensu lato', 'Human endogenous retrovirus K', 'Pandoravirus salinus', 'Macacine herpesvirus 1']

['Enterobacteria phage phiX174 sensu lato', 'Human endogenous retrovirus K113', 'Saccharomyces cerevisiae killer virus M1', 'Alphapapillomavirus 7', 'Primula malacoides virus China/Mar2007']

(288, 294)

TCGA-ZX-AA5X-01A

['Enterobacteria phage phiX174 sensu lato', 'Alphapapillomavirus 9', 'Pandoravirus salinus', 'Simbu virus', 'Human endogenous retrovirus K']

['Enterobacteria phage phiX174 sensu lato', 'Human endogenous retrovirus K113', 'Brome mosaic virus', 'Encephalomyocarditis virus', 'Saccharomyces cerevisiae killer virus M1']

(148, 174)

TCGA-JX-A3Q0-01A

['Enterobacteria phage phiX174 sensu lato', 'Alphapapillomavirus 9', 'Pandoravirus salinus', 'Human endogenous retrovirus K', 'Simbu virus']

['Enterobacteria phage phiX174 sensu lato', 'Human endogenous retrovirus K113', 'Bovine viral diarrhea virus 1', 'Saccharomyces cerevisiae killer virus M1', 'Ball python nidovirus']

(40, 99)

TCGA-FU-A2QG-01A

['Alphapapillomavirus 9', 'Enterobacteria phage phiX174 sensu lato', 'Pandoravirus salinus', 'Human endogenous retrovirus K', 'Tupaiid herpesvirus 1']

['Human papillomavirus type 16', 'Enterobacteria phage phiX174 sensu lato', 'Human endogenous retrovirus K113', 'Saccharomyces cerevisiae killer virus M1', 'White clover cryptic virus 2']

(248, 199)

TCGA-VS-A8QF-01A

['Enterobacteria phage phiX174 sensu lato', 'Alphapapillomavirus 9', 'Human endogenous retrovirus K', 'Pandoravirus salinus', 'Tupaiid herpesvirus 1']

['Enterobacteria phage phiX174 sensu lato', 'Human papillomavirus type 16', 'Human endogenous retrovirus K113', 'Encephalomyocarditis virus', 'Saccharomyces cerevisiae killer virus M1']

(119, 27)

TCGA-C5-A1MK-01A

['Enterobacteria phage phiX174 sensu lato', 'Alphapapillomavirus 9', 'Simbu virus', 'Choristoneura occidentalis granulovirus', 'Pandoravirus salinus']

['Enterobacteria phage phiX174 sensu lato', 'Human endogenous retrovirus K113', 'Saccharomyces cerevisiae killer virus M1', 'White clover cryptic virus 2', 'Primula malacoides virus China/Mar2007']

(129, 10)

TCGA-C5-A1MF-01A

['Enterobacteria phage phiX174 sensu lato', 'Simbu virus', 'Shamonda virus', 'Choristoneura occidentalis granulovirus', 'Pandoravirus salinus']

['Enterobacteria phage phiX174 sensu lato', 'Red clover cryptic virus 2', 'Saccharomyces cerevisiae killer virus M1', 'White clover cryptic virus 2', 'Human endogenous retrovirus K113']

(259, 287)

TCGA-VS-A9UR-01A

['Enterobacteria phage phiX174 sensu lato', 'Alphapapillomavirus 9', 'Human endogenous retrovirus K', 'Pandoravirus salinus', 'Shamonda virus']

['Enterobacteria phage phiX174 sensu lato', 'Human papillomavirus type 16', 'Human endogenous retrovirus K113', 'Primula malacoides virus China/Mar2007', 'Saccharomyces cerevisiae killer virus M1']

(159, 151)

TCGA-EX-A69M-01A

['Enterobacteria phage phiX174 sensu lato', 'Alphapapillomavirus 9', 'Human endogenous retrovirus K', 'Pandoravirus salinus', 'Choristoneura occidentalis granulovirus']

['Enterobacteria phage phiX174 sensu lato', 'Human endogenous retrovirus K113', 'Saccharomyces cerevisiae killer virus M1', 'Gentian ovary ring-spot virus', 'Primula malacoides virus China/Mar2007']

(142, 3)

TCGA-DR-A0ZM-01A

['Enterobacteria phage phiX174 sensu lato', 'Simbu virus', 'Pandoravirus salinus', 'Shamonda virus', 'Choristoneura occidentalis granulovirus']

['Enterobacteria phage phiX174 sensu lato', 'Human endogenous retrovirus K113', 'White clover cryptic virus 2', 'Saccharomyces cerevisiae killer virus M1', 'Red clover cryptic virus 2']

(211, 253)

TCGA-VS-A94Z-01A

['Enterobacteria phage phiX174 sensu lato', 'Human endogenous retrovirus K', 'Alphapapillomavirus 9', 'Pandoravirus salinus', 'Rosellinia necatrix partitivirus 2']

['Enterobacteria phage phiX174 sensu lato', 'Human papillomavirus type 16', 'Human endogenous retrovirus K113', 'Primula malacoides virus China/Mar2007', 'Bovine viral diarrhea virus 1']

(184, 197)

TCGA-C5-A7UC-01A

['Enterobacteria phage phiX174 sensu lato', 'Alphapapillomavirus 7', 'Simbu virus', 'Shamonda virus', 'Pandoravirus salinus']

['Enterobacteria phage phiX174 sensu lato', 'Human endogenous retrovirus K113', 'Woolly monkey sarcoma virus', 'Encephalomyocarditis virus', 'Bovine viral diarrhea virus 1']

(290, 293)

TCGA-VS-A9U5-01A

['Enterobacteria phage phiX174 sensu lato', 'Human endogenous retrovirus K', 'Alphapapillomavirus 9', 'Pandoravirus salinus', 'Choristoneura occidentalis granulovirus']

['Enterobacteria phage phiX174 sensu lato', 'Human papillomavirus type 16', 'Human endogenous retrovirus K113', 'Bovine viral diarrhea virus 1', 'Saccharomyces cerevisiae killer virus M1']

(77, 82)

TCGA-IR-A3LC-01A

['Enterobacteria phage phiX174 sensu lato', 'Simbu virus', 'Alphapapillomavirus 9', 'Shamonda virus', 'Choristoneura occidentalis granulovirus']

['Enterobacteria phage phiX174 sensu lato', 'Human papillomavirus type 16', 'Human endogenous retrovirus K113', 'Primula malacoides virus China/Mar2007', 'Saccharomyces cerevisiae killer virus M1']

(24, 122)

TCGA-EA-A50E-01A

['Enterobacteria phage phiX174 sensu lato', 'Pandoravirus salinus', 'Human endogenous retrovirus K', 'Simbu virus', 'Shamonda virus']

['Enterobacteria phage phiX174 sensu lato', 'Human papillomavirus type 16', 'Human endogenous retrovirus K113', 'White clover cryptic virus 2', 'Hepatitis C virus']

(75, 78)

TCGA-IR-A3LH-01A

['Enterobacteria phage phiX174 sensu lato', 'Simbu virus', 'Shamonda virus', 'Choristoneura occidentalis granulovirus', 'Alphapapillomavirus 7']

['Enterobacteria phage phiX174 sensu lato', 'Human endogenous retrovirus K113', 'Saccharomyces cerevisiae killer virus M1', 'White clover cryptic virus 2', 'Primula malacoides virus China/Mar2007']

(137, 4)

TCGA-C5-A1BM-01A

['Enterobacteria phage phiX174 sensu lato', 'Pandoravirus salinus', 'Shamonda virus', 'Simbu virus', 'Tupaiid herpesvirus 1']

['Enterobacteria phage phiX174 sensu lato', 'Alphapapillomavirus 7', 'Human endogenous retrovirus K113', 'Primula malacoides virus China/Mar2007', 'Saccharomyces cerevisiae killer virus M1']

(6, 144)

TCGA-JW-A5VL-01A

['Enterobacteria phage phiX174 sensu lato', 'Alphapapillomavirus 9', 'Human endogenous retrovirus K', 'Pandoravirus salinus', 'Choristoneura occidentalis granulovirus']

['Enterobacteria phage phiX174 sensu lato', 'Human papillomavirus type 16', 'Human endogenous retrovirus K113', 'Primula malacoides virus China/Mar2007', 'Saccharomyces cerevisiae killer virus M1']

(131, 15)

TCGA-C5-A1ME-01A

['Enterobacteria phage phiX174 sensu lato', 'Choristoneura occidentalis granulovirus', 'Human endogenous retrovirus K', 'Simbu virus', 'Shamonda virus']

['Enterobacteria phage phiX174 sensu lato', 'Alphapapillomavirus 7', 'Human endogenous retrovirus K113', 'White clover cryptic virus 2', 'Saccharomyces cerevisiae killer virus M1']

(34, 96)

TCGA-FU-A3WB-01A

['Alphapapillomavirus 9', 'Enterobacteria phage phiX174 sensu lato', 'Pandoravirus salinus', 'Human endogenous retrovirus K', 'White spot syndrome virus']

['Enterobacteria phage phiX174 sensu lato', 'Human papillomavirus type 16', 'Human endogenous retrovirus K113', 'Saccharomyces cerevisiae killer virus M1', 'White clover cryptic virus 2']

(100, 48)

TCGA-EK-A2RL-01A

['Enterobacteria phage phiX174 sensu lato', 'Alphapapillomavirus 9', 'Human endogenous retrovirus K', 'Shamonda virus', 'Simbu virus']

['Enterobacteria phage phiX174 sensu lato', 'Human papillomavirus type 16', 'Human endogenous retrovirus K113', 'Red clover cryptic virus 2', 'Primula malacoides virus China/Mar2007']

(198, 230)

TCGA-VS-A8EC-01A

['Enterobacteria phage phiX174 sensu lato', 'Alphapapillomavirus 9', 'Human endogenous retrovirus K', 'Pandoravirus salinus', 'Shamonda virus']

['Enterobacteria phage phiX174 sensu lato', 'Human papillomavirus type 16', 'Human endogenous retrovirus K113', 'Encephalomyocarditis virus', 'Bovine viral diarrhea virus 1']

(112, 21)

TCGA-C5-A1BF-01B

['Enterobacteria phage phiX174 sensu lato', 'Choristoneura occidentalis granulovirus', 'Shamonda virus', 'Pandoravirus salinus', 'Pandoravirus dulcis']

['Enterobacteria phage phiX174 sensu lato', 'Alphapapillomavirus 7', 'Human endogenous retrovirus K113', 'Primula malacoides virus China/Mar2007', 'Red clover cryptic virus 2']

(58, 107)

TCGA-FU-A23L-01A

['Enterobacteria phage phiX174 sensu lato', 'Alphapapillomavirus 7', 'Human endogenous retrovirus K', 'Pandoravirus salinus', 'Simbu virus']

['Enterobacteria phage phiX174 sensu lato', 'Human endogenous retrovirus K113', 'Saccharomyces cerevisiae killer virus M1', 'Primula malacoides virus China/Mar2007', 'White clover cryptic virus 2']

(109, 17)

TCGA-EX-A1H5-01A

['Enterobacteria phage phiX174 sensu lato', 'Alphapapillomavirus 9', 'Simbu virus', 'Pandoravirus salinus', 'Shamonda virus']

['Enterobacteria phage phiX174 sensu lato', 'Human papillomavirus type 16', 'Human endogenous retrovirus K113', 'Saccharomyces cerevisiae killer virus M1', 'Primula malacoides virus China/Mar2007']

(134, 12)

TCGA-BI-A0VS-01A

['Enterobacteria phage phiX174 sensu lato', 'Human endogenous retrovirus K', 'Simbu virus', 'Pandoravirus salinus', 'Alphapapillomavirus 9']

['Enterobacteria phage phiX174 sensu lato', 'Human papillomavirus type 16', 'Human endogenous retrovirus K113', 'Primula malacoides virus China/Mar2007', 'Saccharomyces cerevisiae killer virus M1']

(302, 302)

TCGA-VS-A9U6-01A

['Enterobacteria phage phiX174 sensu lato', 'Human endogenous retrovirus K', 'Alphapapillomavirus 9', 'Pandoravirus salinus', 'Glypta fumiferanae ichnovirus']

['Enterobacteria phage phiX174 sensu lato', 'Human endogenous retrovirus K113', 'Bovine viral diarrhea virus 1', 'Y73 sarcoma virus', 'Primula malacoides virus China/Mar2007']

(214, 244)

TCGA-MA-AA41-01A

['Enterobacteria phage phiX174 sensu lato', 'Alphapapillomavirus 9', 'Human endogenous retrovirus K', 'Pandoravirus salinus', 'Macacine herpesvirus 1']

['Enterobacteria phage phiX174 sensu lato', 'Human papillomavirus type 16', 'Human endogenous retrovirus K113', 'Encephalomyocarditis virus', 'Saccharomyces cerevisiae killer virus M1']

(235, 211)

TCGA-C5-A907-01A

['Enterobacteria phage phiX174 sensu lato', 'Alphapapillomavirus 7', 'Human endogenous retrovirus K', 'Pandoravirus salinus', 'Tupaiid herpesvirus 1']

['Enterobacteria phage phiX174 sensu lato', 'Alphapapillomavirus 7', 'Human endogenous retrovirus K113', 'Encephalomyocarditis virus', 'Bovine viral diarrhea virus 1']
